# Efficacy and safety profile of angiotensin receptor neprilysin inhibitors in the management of heart failure: a systematic review and meta-analysis of randomized controlled trials

**DOI:** 10.1007/s10741-022-10273-3

**Published:** 2022-10-03

**Authors:** Juan Gao, Cong Zhao, Wen-Zhong Zhang, Song Liu, Hui Xin, Zhe-Xun Lian

**Affiliations:** Department of Cardiology, The Affiliated Hospital of Qingdao University, Qingdao, China

**Keywords:** Ejection fraction, Heart failure, Meta-analysis, Sacubitril–valsartan

## Abstract

Several guidelines have recommended the use of angiotensin receptor neprilysin inhibitors (ARNIs) as replacement for angiotensin-converting enzyme inhibitors in the management of heart failure. Till date, there are no reviews done that comprehensively cover different aspects of efficacy and safety parameters. Hence, we have performed a comprehensive systematic review and meta-analysis on role of ARNIs for the management of heart failure patients. Searches were done in Embase, Scopus, China National Knowledge Infrastructure, Chinese Biomedical Literature Database, PubMed Central, Cochrane Library, MEDLINE, Google Scholar, ScienceDirect and Clinicaltrials.gov until June 2022. Risk of bias assessment was done with Cochrane’s risk of bias tool. Meta-analysis was carried out using random-effects model. Pooled standardized mean difference (SMD)/mean difference (MD) and/or risk ratio (RR) with 95% confidence intervals (CIs) was reported. In total, we analysed 34 studies, with almost all of them had a high risk of bias. Pooled RR was 0.88 (95% CI: 0.82–0.95) for all-cause mortality, 0.84 (95% CI: 0.77–0.92) for cardiovascular mortality and 0.78 (95% CI: 0.70–0.87) for hospitalization. Pooled MD was 3.74 (95% CI: 1.93–5.55) for left ventricular ejection fraction, −2.16 (95% CI: −3.58 to −0.74) for left atrial volume index, −3.80 (95% CI: −6.60 to −1.00) for left ventricular end-diastolic dimension and −1.16 (95% CI: −1.98 to −0.35) for *E*/*E*′ ratio. Regarding adverse events, pooled RR was 1.55 (95% CI: 1.31–1.85) for symptomatic hypotension, 0.93 (95% CI: 0.78–1.11) for worsening renal function, 1.09 (95% CI: 0.94–1.26) for hyperkalaemia and 1.29 (95% CI: 0.67–2.50) for angioedema. ARNIs had beneficial efficacy and safety profile on the management of heart failure especially patients with reduced ejection fraction.

**Supplementary Information:**

The online version contains supplementary material available at 10.1007/s10741-022-10273-3.

## Introduction

Heart failure was reported as an emerging epidemic almost three decades ago [1]. Globally, the current burden of heart failure is estimated to be nearly 64 million [1]. The prevalence of heart failure was found to be more than 1% across various countries and regions around the world [2]. It is ever growing over the past decade across both developed and developing countries [2]. Moreover, this can increase the economic burden of all the countries in the world [3].

Heart failure is a clinical condition affecting ejection function and/or ventricular filling caused by several cardiac functional or structural diseases [3]. It acts as an end-stage disease across various forms of cardiovascular diseases (CVDs), making it known as “last battlefield” of the CVDs [4, 5]. Several medications have been prescribed to manage heart failure, like beta blockers, angiotensin-converting enzyme (ACE) inhibitors, calcium channel blockers (CCBs) and angiotensin receptor blockers (ARBs), without any major success in terms of efficacy [6–8].

Angiotensin receptor neprilysin inhibitor (ARNI), i.e. sacubitril–valsartan, has been tried to treat the patients with heart failure [9]. Neprilysin damages the biologically active natriuretic peptide, including the atrial, B-type and C-type natriuretic peptides, but not biologically inert natriuretic pro-hormone B-type natriuretic peptide (NT-proBNP), which is not a substrate for these enzymes [10]. By augmenting active NT peptides, inhibition of neprilysin increases the generation of the myocardial cyclic guanosine monophosphate (cGMP), which improves the myocardial relaxation and hypertrophy reduction [11, 12].

The European Society of Cardiology, American College of Cardiology/American Heart Association Task Force on Clinical Practice Guidelines and the Heart Failure Society of America have recommended ARNIs as replacement for the ACE inhibitors in the management of heart failure especially those with reduced ejection fraction remaining symptomatic and belonging to the New York Heart Association (NYHA) classes II to IV [13, 14]. However, only fewer systematic reviews are available on demonstrating the improvement in cardiac function after the ARNI therapy in patients with heart failure [15–18]. The available reviews have also included only a limited number of trials or fewer outcomes with respect to the efficacy and safety of ARNIs. Till date, there are no reviews done on this topic that comprehensively cover different aspects of efficacy and safety parameters, and the available primary studies have provided some inconclusive evidences. Hence, we have performed a comprehensive systematic review and meta-analysis on the role of ARNIs for the management of heart failure patients.

## Materials and methods

### Eligibility criteria

#### Study design

Randomized controlled trials (RCTs) were eligible for inclusion. Full-text studies that are eligible were included while the case reports/series/unpublished grey literature was excluded from the study.

#### Study participants

Studies containing the heart failure patients were incorporated irrespective of their age and gender, comorbidity, status of ejection fraction and acute state or chronicity of the condition. Separate analysis based on these characteristics was tried during analysis stage.

#### Intervention and comparator group

Studies assessing the effectiveness of ARNI, i.e. sacubitril–valsartan, compared to placebo or control or any other medications were eligible for inclusion irrespective of the frequency or duration of intervention.

#### Outcome

Efficacy parameters include the following: all-cause mortality, cardiovascular mortality, hospitalization, quality of life and improvement in NYHA functional status.

Echocardiographic parameters are as follows: left ventricular ejection fraction (LVEF), left atrial volume index (LAVI), left ventricular end-diastolic dimension (LVED) and early filling (*E*)-to-early diastolic mitral annular velocity (*E*′) (*E*/*E*′) ratio.

Arrhythmia endpoints include the following: atrial arrhythmias (atrial fibrillation and/or atrial flutter) and ventricular arrhythmias (ventricular fibrillation and/or ventricular tachycardia).

Adverse events include symptomatic hypotension, worsening of renal function, hyperkalaemia and angioedema.

Blood parameter includes N-terminal pro-hormone of brain natriuretic peptide (NT-proBNP).

### Search strategy

An extensive, systematic and comprehensive literature review was done by executing the search in various databases such as Embase, Scopus, China National Knowledge Infrastructure (CNKI), Chinese Biomedical Literature Database, PubMed Central, Cochrane Library, MEDLINE, Google Scholar, ScienceDirect and Clinicaltrials.gov. For the purpose of carrying out our search strategy, we have merged free-text headings and medical topic headings (MeSH). Using the appropriate Boolean operators (“AND”, “OR”, “NOT”) in between the pre-defined search phrases, we carried out the search strategy. The search terms utilized during the search are provided in the Supplementary Appendix. The following additional filters were applied during the process of literature search: time point (January 1964, i.e. inception of databases till June 2022) and no language filters.

### Steps in study selection

The initial stage of the study selection procedure involved two independent researchers (JG and ZXL) reviewing the title, keywords and abstract. The full-text papers were retrieved by each of the two investigators, who then shortlisted them for the second round of screening based on the eligibility requirements. By coming to an agreement, the two investigators were able to settle any disagreements that arose during the initial screening stage. The second phase involved the screening of the recovered full-text studies by the two researchers (JG and ZXL), who ultimately included those that met the eligibility requirements and underwent additional analysis based on these studies. *Preferred Reporting Items for Systematic Reviews and Meta-Analyses (PRISMA) checklist 2020* was used for reporting this review [19].

### Data extraction procedure

Both investigators (CZ and ZWZ) participated in the manual data extraction procedure utilising a pre-specified semi-structured data collection form that was established at the stage of the protocol itself after deciding which full-text publications were suitable for inclusion and analysis in the review. The following details were gathered: the names of the authors, the study’s title, the year it was published and the year it was conducted, the length of the study, its design, its setting, its country or region, its sample size, the outcome assessment tool and other information, the participants’ ages on average, the specifics of its randomization, their qualifications, its quality-related information and its outcome-related information. The second author (CZ) entered the data, and the third author double-checked the entry’s accuracy before it was recorded (ZWZ).

### Risk of bias (quality) assessment

Two investigators (SL and HX) were responsible for assessing the quality of included studies. They have used the RoB2 tool, i.e. “Cochrane risk of bias tool for RCTs” [20]. The tool assessed bias risk based on randomization, deviation from intended intervention, missing data, outcome measurement and selective reporting of results. Depending on the response, each study was identified to have low, high or some concerns with respect to bias risk.

### Statistical analysis

All the analysis was performed using Stata version 14.2. For outcomes that were continuous in nature, mean, standard deviation (SD) and total sample size were obtained for both groups. The pooled effect was calculated as mean difference (MD) or standardized mean difference (SMD, for outcomes like quality of life as each study uses different scales for assessment) with 95% confidence interval (CI), depending on the outcome. For binary outcomes, frequency of events and participants in intervention and control arm were entered and pooled estimate was obtained as risk ratio (RR) along with the 95% CI. Visual representation of these pooled estimates was done by forest plot. Random-effects model with inverse variance method was utilized to account for methodological heterogeneity [21].

Heterogeneity was evaluated by the chi-square of heterogeneity and *I*^2^ statistic. *p* value less than 0.05 in chi-square test indicates significant heterogeneity, while *I*^2^ value was used to quantify the heterogeneity [21]. Subgroup analysis and meta-regression were performed to investigate the outcomes with substantial heterogeneity for the following variables: country/study region, follow-up duration, dose of sacubitril–valsartan and type of control group. Publication bias assessment was done using Egger’s test and depicted visually by funnel plot. Egger’s test *p* value less than 0.05 or asymmetrical funnel plot indicates the possibility of the presence of publication bias.

## Results

### Study selection

**Fig. 1:**
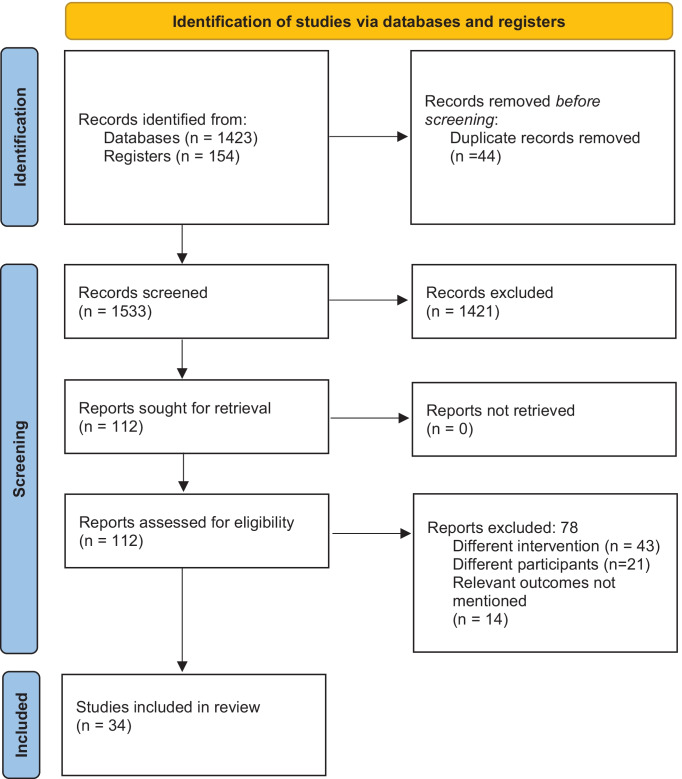
Search strategy

In primary screening, we retrieved 108 full-text studies, which, after removal of duplicates, become 103 studies. These studies undergone secondary screening in addition to the three articles retrieved from the bibliography of the screened articles. Finally, we included data from 34 studies satisfying the inclusion criteria (Fig. 1) [22–55].

### Study characteristics

Only RCTs were included in the review. Most studies (17 out of 34 studies) were conducted in China, followed by United States (US) and multi-country studies. The mean age of study participants in the intervention arm ranged from 53 to 74.4 years, while that in the control arm ranged from 55 to 75.9 years. The sample sizes amongst the included studies varied from 16 to 4187 in the intervention arm and 15 to 4212 in the control arm (Table 1).

**Table 1:** Characteristics of the included studies (*N* = 34)

**Author and year**	**Country**	**Heart failure type**	**Acute or chronic**	**Intervention group**	**Control group**	**Sample size in I vs C arm**	**Follow-up duration (in months)**	**Mean age (in years)**
Bano et al. (2021) [22]	Pakistan	HFrEF	Chronic	Sacubitril–valsartan 50 mg or 100 mg twice daily	Enalapril 2.5 mg or 5 mg twice daily	*I* = 181 *C* = 183	12	*I* = 53 *C* = 55
CLCZ696B2223 (2013)	NR	HFrEF	Chronic	Sacubitril–valsartan 200 mg twice daily	Valsartan 160 mg twice daily	*I* = 16 *C* = 15	7 days	NR
CLCZ696BDE01 (2019)	NR	HFrEF	Chronic	Sacubitril–valsartan 100 mg twice daily	Enalapril 5 mg twice daily	*I* = 103 *C* = 98	3	NR
Chai et al. (2019) [42]	China	HFrEF	Chronic	Sacubitril–valsartan 50 mg twice daily	Milinon	*I* = 40 *C* = 40	3	NR
Chen et al. (2020) [41]	China	HFmEF	Chronic	Sacubitril–valsartan 200 mg twice daily	ACEI/ARB	*I* = 53 *C* = 53	6	*I* = 72.3 *C* = 69.5
Dai et al. (2019) [43]	China	HFrEF	Chronic	Sacubitril–valsartan (dose NR)	Ramipril	*I* = 98 *C* = 98	6	NR
Desai et al. (2019) [44] [EVALUATE HF]	USA	HFrEF	Chronic	Sacubitril–valsartan 200 mg twice daily	Enalapril 10 mg twice daily	*I* = 231 *C* = 233	2.75	*I* = 67.8 *C* = 66.7
Du et al. (2022) [23]	China	HFrEF	Chronic	Sacubitril–valsartan 100 mg twice daily	Valsartan 80 mg twice daily	*I* = 30 *C* = 30	6	*I* = 74.4 *C* = 75.9
Gao et al. (2019) [45]	China	HFrEF	Chronic	Sacubitril–valsartan (dose NR)	Valsartan	*I* = 17 *C* = 17	1.75	NR
Hao et al. (2019) [46]	China	HFrEF	Chronic	Sacubitril–valsartan 200 mg twice daily	Valsartan 160 mg twice daily	*I* = 30 *C* = 30	1.75	NR
Huang et al. (2019) [24]	China	HFpEF	Chronic	Sacubitril–valsartan (dose NR)	Usual care	*I* = 39 *C* = 38	6	NR
Kang et al. (2019) [25] [PRIME]	Korea	HFrEF	Chronic	Sacubitril–valsartan 200 mg twice daily	Valsartan 40 to 80 mg twice daily	*I* = 60 *C* = 58	12	*I* = 64.7 *C* = 60.5
Khandwalla et al. (2020) [26] [AWAKE HF]	USA	HFrEF	Chronic	Sacubitril–valsartan 200 mg twice daily	Enalapril 10 mg twice daily	*I* = 69 *C* = 70	4	*I* = 62.3 *C* = 64.2
Li et al. (2019a) [27] (1)	China	HFrEF	Chronic	Sacubitril–valsartan (dose NR)	Enalapril	*I* = 62 *C* = 64	6	NR
Li et al. (2019b) [28] (2)	China	NR	Chronic	Sacubitril–valsartan 100 mg twice daily	Enalapril 10 mg twice daily	*I* = 47 *C* = 49	12	NR
Li et al. (2021) [29]	China	HFrEF	Chronic	Sacubitril–valsartan 50 mg twice daily	Perindopril 4 mg once daily	*I* = 40 *C* = 40	3	*I* = 63.2 *C* = 62.8
Liang (2022) [53]	China	HFrEF	Chronic	Sacubitril–valsartan (dose NR)	Conventional anti-HF treatment	*I* = 60 *C* = 60	NR	NA
Mann et al. (2022) [30] [LIFE]	USA	HFrEF	Chronic	Sacubitril–valsartan 200 mg twice daily	Valsartan 160 mg twice daily	*I* = 167 *C* = 168	6	*I* = 60.2 *C* = 58.3
McMurray et al. (2014) [31] [PARADIGM HF]	47 countries	HFrEF	Chronic	Sacubitril–valsartan 200 mg twice daily	Enalapril 10 mg twice daily	*I* = 4187 *C* = 4212	27	*I* = 63.8 *C* = 63.8
Mukhamedova et al. (2022) [55]	Uzbekistan	HFrEF	Chronic	Sacubitril–valsartan (dose NR)	Valsartan	*I* = 60 *C* = 60	NR	58.2
OUTSTEP HF (2019)	19 European countries	HFrEF	Chronic	Sacubitril–valsartan 200 mg twice daily	Enalapril 10 mg twice daily	*I* = 309 *C* = 310	4	*I* = 67.2 *C* = 66.6
Pieske et al. (2021) [51] [PARALLAX HF]	32 countries	HFpEF	Chronic	Sacubitril–valsartan 200 mg twice daily	Enalapril 10 mg/valsartan 160 mg/placebo	*I* = 1286 *C* = 1286	6	*I* = 72.9 *C* = 72.4
Qin et al. (2022) [32]	China	HFrEF	Chronic	Sacubitril–valsartan 100 mg twice daily	Valsartan 160 mg twice daily	*I* = 36 *C* = 36	2	*I* = 66.2 *C* = 67.5
Qu (2022) [52]	China	HFrEF	Chronic	Sacubitril–valsartan (dose NR)	Benazepril	*I* = 50 *C* = 50	NR	NR
Shi et al. (2020) [40]	China	HFpEF	Chronic	Sacubitril–valsartan 100 mg twice daily	Valsartan 80 mg twice daily	*I* = 20 *C* = 22	3	*I* = 68.5 *C* = 66.7
Solomon et al. (2012) [33] [PARAMOUNT]	13 countries	HFpEF	Chronic	Sacubitril–valsartan 200 mg twice daily	Valsartan 160 mg twice daily	*I* = 149 *C* = 152	21	*I* = 70.9 *C* = 71.2
Solomon et al. (2019) [34] [PARAGON HF]	43 countries	HFpEF	Chronic	Sacubitril–valsartan 200 mg twice daily	Valsartan 160 mg twice daily	*I* = 2407 *C* = 2389	35	*I* = 72.7 *C* = 72.8
Tsutsui et al. (2021) [35] [PARALLEL HF]	Japan	HFrEF	Chronic	Sacubitril–valsartan 200 mg twice daily	Enalapril 10 mg twice daily	*I* = 111 *C* = 112	33.9	*I* = 69 *C* = 66.7
Tumasyan et al. (2019) [47]	NR	HFmEF	Chronic	Sacubitril–valsartan 200 mg twice daily	Valsartan 160 mg + spironolactone 25 mg	*I* = 26 *C* = 53	12	NR
Velazquez et al. (2019) [36] [PIONEER HF]	USA	HFrEF	Acute	Sacubitril–valsartan 200 mg twice daily	Enalapril 10 mg twice daily	*I* = 440 *C* = 441	2	*I* = 61 *C* = 63
Wang et al. (2019) [39]	China	HFmEF	Chronic	Sacubitril–valsartan 200 mg twice daily	ACEI/ARB	*I* = 48 *C* = 48	12	*I* = 55.9 *C* = 55.9
Zhao et al. ( 2022) [37]	China	HFrEF	Chronic	Sacubitril–valsartan 50 mg twice daily	Enalapril 10 mg once daily	*I* = 52 *C* = 45	6	*I* = 68.6 *C* = 66.7
Zhu et al. (2021) [54]	China	HFrEF	Chronic	Sacubitril–valsartan (dose NR)	Benazepril hydrochloride	*I* = 51 *C* = 51	12	NR
dos Santos et al. (2021) [38] [NEPRIExTOL]	Brazil	HFrEF	Chronic	Sacubitril–valsartan 100 mg twice daily	Enalapril 10 mg or 20 mg twice daily	*I* = 26 *C* = 18	6	*I* = 56 *C* = 61

*HFrEF* heart failure with reduced ejection fraction, *HFmEF* heart failure with a mid-range ejection fraction, *HFpEF* heart failure with preserved ejection fraction, *NR* no record

### Risk of bias assessment

Almost one-third of studies (11 out of 34 studies) had a low risk of bias with respect to randomization process and deviation from intended intervention. Only nine studies had a low risk of bias with respect to missing outcome data. Majority of the studies had a high risk of bias with respect to selective reporting of results and measurement of outcomes. Most studies (22 out of 34 studies) had a higher risk of bias (Table 2).

**Table 2:** Risk of bias assessment (*N* = 34)

**S. No.**	**Author and year**	**Randomization process**	**Deviation from intended intervention**	**Missing outcome data**	**Measurement of the outcome**	**Selection of the reported results**	**Overall**
1	Bano et al. (2021) [22]	Low	Low	Low	Some concerns	Some concerns	Some concerns
2	CLCZ696B2223 (2013)	Low	Low	Low	Low	High	Some concerns
3	CLCZ696BDE01 (2019)	Some concerns	Some concerns	Low	Low	Low	Some concerns
4	Chai et al. (2019) [42]	Some concerns	Some concerns	High	High	High	High
5	Chen et al. (2020) [41]	Some concerns	Some concerns	High	High	High	High
6	Dai et al. (2019) [43]	Some concerns	Some concerns	High	High	High	High
7	Desai et al. (2019) [44] [EVALUATE HF]	Low	Low	Some concerns	Some concerns	Low	Some concerns
8	Du et al (2022) [23]	Some concerns	Some concerns	High	High	High	High
9	Gao et al. (2019) [45]	Some concerns	Some concerns	High	High	High	High
10	Hao et al. (2019) [46]	Some concerns	Some concerns	High	High	High	High
11	Huang et al. (2019) [24]	Some concerns	Some concerns	High	High	High	High
12	Kang et al. (2019) [25] [PRIME]	Some concerns	Some concerns	High	Low	Low	High
13	Khandwalla et al. (2020) [26] [AWAKE HF]	Some concerns	Some concerns	High	Low	Low	High
14	Li et al. (2019) [27] (1)	Some concerns	Some concerns	High	High	High	High
15	Li et al. (2019) [28] (2)	Some concerns	Some concerns	High	High	High	High
16	Li et al. (2021) [29]	High	High	High	High	High	High
17	Liang (2022) [53]	Some concerns	High	High	High	High	High
18	Mann et al. (2022) [30] [LIFE]	Low	Low	Low	Some concerns	Low	Some concerns
19	McMurray et al. (2014) [31] [PARADIGM HF]	Low	Low	Low	Low	Low	Low
20	Mukhamedova et al. (2022) [55]	Some concerns	Some concerns	High	High	High	High
21	OUTSTEP HF (2019)	Low	Low	Low	Low	Low	Low
22	Pieske et al. (2021) [51] [PARALLAX HF]	Low	Low	Low	Some concerns	Low	Some concerns
23	Qin et al. (2022) [32]	Some concerns	Some concerns	High	High	High	High
24	Qu (2022) [52]	Some concerns	Some concerns	High	High	High	High
25	Shi et al. (2020) [40]	Some concerns	Some concerns	High	High	High	High
26	Solomon et al. (2012) [33] [PARAMOUNT]	Low	Low	Low	Low	Low	Low
27	Solomon et al. (2019) [34] [PARAGON HF]	Low	Low	Low	Low	Low	Low
28	Tsutsui et al. (2021) [35] [PARALLEL HF]	Some concerns	Some concerns	High	High	High	High
29	Tumasyan et al. (2019) [47]	Some concerns	Some concerns	High	High	High	High
30	Velazquez et al. (2019) [36] [PIONEER HF]	Low	Low	Low	Low	Low	Low
31	Wang (2019) [39]	Some concerns	Some concerns	High	High	High	High
32	Zhao et al. ( 2022) [37]	Some concerns	Some concerns	High	High	High	High
33	Zhu et al. (2021) [54]	Some concerns	Some concerns	High	High	High	High
34	dos Santos et al. (2021) [38] [NEPRIExTOL]	Low	Low	Low	Some concerns	Low	Some concerns

### Efficacy parameters

#### All-cause mortality

**Fig. 2:**
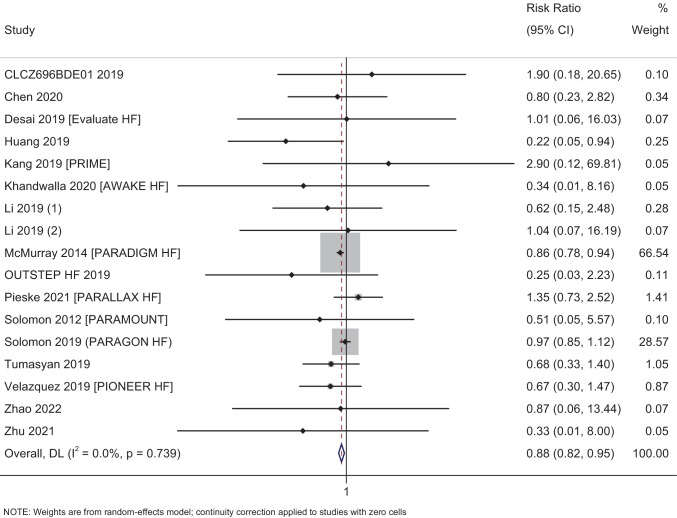
Forest plot showing the difference in all-cause mortality between sacubitril–valsartan and control group patients

In total, 17 studies with 19,176 participants have reported on the efficacy of sacubitril–valsartan on the all-cause mortality amongst heart failure patients. The pooled RR was 0.88 (95% CI: 0.82 to 0.95; *I*^2^ = 0%), indicating that the patients receiving sacubitril–valsartan had a significantly lower risk of having all-cause mortality when compared to patients receiving standard care or placebo (*p* = 0.001) (Fig. 2).

Subgroup analysis based on the status of ejection fraction revealed that the patients with reduced ejection fraction had a significant reduction in all-cause mortality (pooled RR = 0.85; 95% CI: 0.78–0.93; *p* < 0.001), while patients with preserved or medium ejection fraction had non-significant reduction in all-cause mortality (pooled RR = 0.91; 95% CI: 0.67–1.22; *p* = 0.52) following administration of sacubitril–valsartan (Supplementary Fig. 1). Subgroup analysis based on the control group showed that the sacubitril–valsartan showed maximum efficacy against placebo or usual care arm (pooled RR = 0.22; 95% CI: 0.05–0.94; *p* = 0.04) followed by ACE inhibitors (pooled RR = 0.85; 95% CI: 0.78–0.93; *p* < 0.001), while it was non-significant against ARBs (pooled RR = 0.96; 95% CI: 0.84–1.10; *p* = 0.56) (Supplementary Fig. 2). Only one study was conducted amongst acute heart failure patients while the rest of the studies are conducted amongst chronic heart failure patients. Hence, subgroup analysis based on the duration of heart failure could not be conducted for any of the outcomes. Subgroup analysis based on the dose of sacubitril–valsartan could not be performed as one study each has used a dose of 50 mg and 100 mg, while the rest of the studies used a dose of 200 mg twice daily.

Assessment of publication bias revealed a symmetrical funnel plot with non-significant Egger’s test (*p* = 0.30), indicating the absence of publication bias (Supplementary Fig. 3). Meta-regression was not performed as there was no statistical heterogeneity for the all-cause mortality outcome.

#### Cardiovascular mortality

**Fig. 3:**
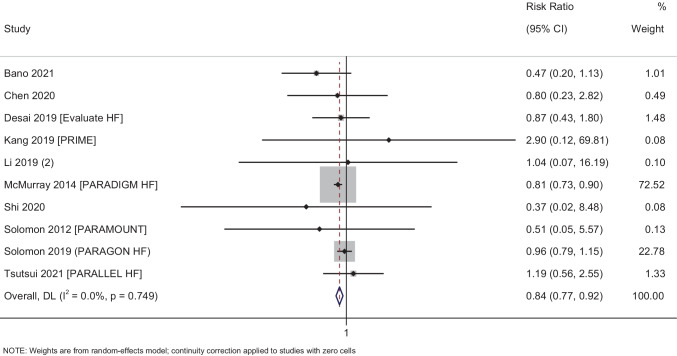
Forest plot showing the difference in cardiovascular mortality between sacubitril–valsartan and control group patients

In total, 10 studies with 14,909 participants have reported on the efficacy of sacubitril–valsartan on the cardiovascular mortality amongst heart failure patients. The pooled RR was 0.84 (95% CI: 0.77 to 0.92; *I*^2^ = 0%), indicating that the patients receiving sacubitril–valsartan had a significantly lower risk of having cardiovascular mortality when compared to patients receiving any other medications (*p* < 0.001) (Fig. 3).

Subgroup analysis based on the status of ejection fraction revealed that the patients with reduced ejection fraction had a significant reduction in cardiovascular mortality (pooled RR = 0.81; 95% CI: 0.73–0.90; *p* < 0.001), while patients with preserved or medium ejection fraction had non-significant reduction in all-cause mortality (pooled RR = 0.95; 95% CI: 0.79–1.13; *p* = 0.54) following administration of sacubitril–valsartan (Supplementary Fig. 4). Subgroup analysis based on the control group showed that the sacubitril–valsartan showed maximum efficacy against ACE inhibitors (pooled RR = 0.81; 95% CI: 0.73–0.90; *p* < 0.001), while it was non-significant against ARBs (pooled RR = 0.95; 95% CI: 0.79–1.14; *p* = 0.60) (Supplementary Fig. 5). Subgroup analysis based on the dose of sacubitril–valsartan could not be performed as almost all the studies for this outcome used a dose of 200 mg twice daily.

Assessment of publication bias revealed a symmetrical funnel plot with non-significant Egger’s test (*p* = 0.50), indicating the absence of publication bias (Supplementary Fig. 6). Meta-regression was not performed as there was no statistical heterogeneity for the cardiovascular mortality outcome.

#### Hospitalization

**Fig. 4:**
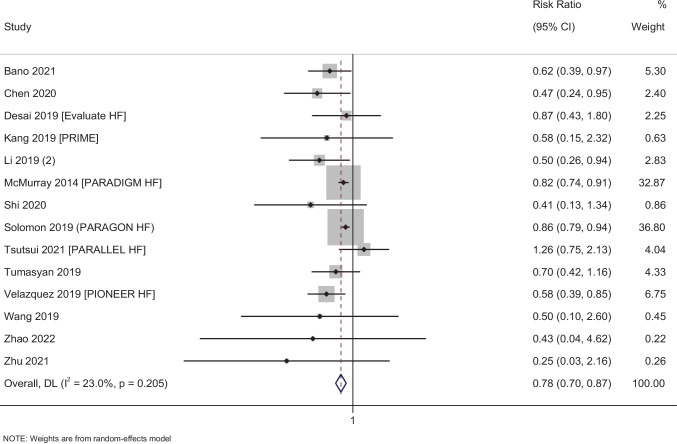
Forest plot showing the difference in hospitalization between sacubitril–valsartan and control group patients

In total, 14 studies with 15,866 participants have reported on the efficacy of sacubitril–valsartan on the rate of hospitalization amongst heart failure patients. The pooled RR was 0.78 (95% CI: 0.70 to 0.87; *I*^2^ = 23%), indicating that the patients receiving sacubitril–valsartan had a significantly lower risk of having hospitalizations when compared to patients receiving any other medications (*p* < 0.001) (Fig. 4).

Subgroup analysis based on the status of ejection fraction revealed that the patients with either reduced ejection fraction or preserved ejection fraction had a significant reduction in hospitalizations following the administration of sacubitril–valsartan (Supplementary Fig. 7). Subgroup analysis based on the control group also showed that the administration of sacubitril–valsartan was efficacious against ACE inhibitors and ARBs for hospitalizations (Supplementary Fig. 8). Subgroup analysis based on the dose of sacubitril–valsartan could not be performed as almost all the studies for this outcome used a dose of 200 mg twice daily.

Assessment of publication bias revealed an asymmetrical funnel plot with significant Egger’s test (*p* = 0.04), indicating the presence of publication bias (Supplementary Fig. 9). Meta-regression was not performed as there was only mild statistical heterogeneity for the hospitalization outcome.

#### Quality of life

In total, 3 studies with 3080 participants have reported on the efficacy of sacubitril–valsartan on the quality of life amongst heart failure patients. The pooled SMD was 0.04 (95% CI: − 0.03 to 0.11; *I*^2^ = 0%), indicating no significant difference between sacubitril–valsartan and control group patients in terms of quality of life (*p* = 0.23) (Supplementary Fig. 10). Subgroup analysis and publication bias assessment could not be performed due to limitation in the number of studies.

#### Improvement in NYHA functional status

In total, 6 studies with 7854 participants have reported on the efficacy of sacubitril–valsartan on the improvement in NYHA functional status amongst heart failure patients. The pooled RR was 1.21 (95% CI: 0.99 to 1.47; *I*^2^ = 58.9%), indicating no significant difference between sacubitril–valsartan and control group patients in terms of improvement in NYHA functional status (*p* = 0.06) (Supplementary Fig. 11). Subgroup analysis and publication bias assessment could not be performed due to limitation in the number of studies.

### Echocardiographic parameters

#### LVEF

**Fig. 5:**
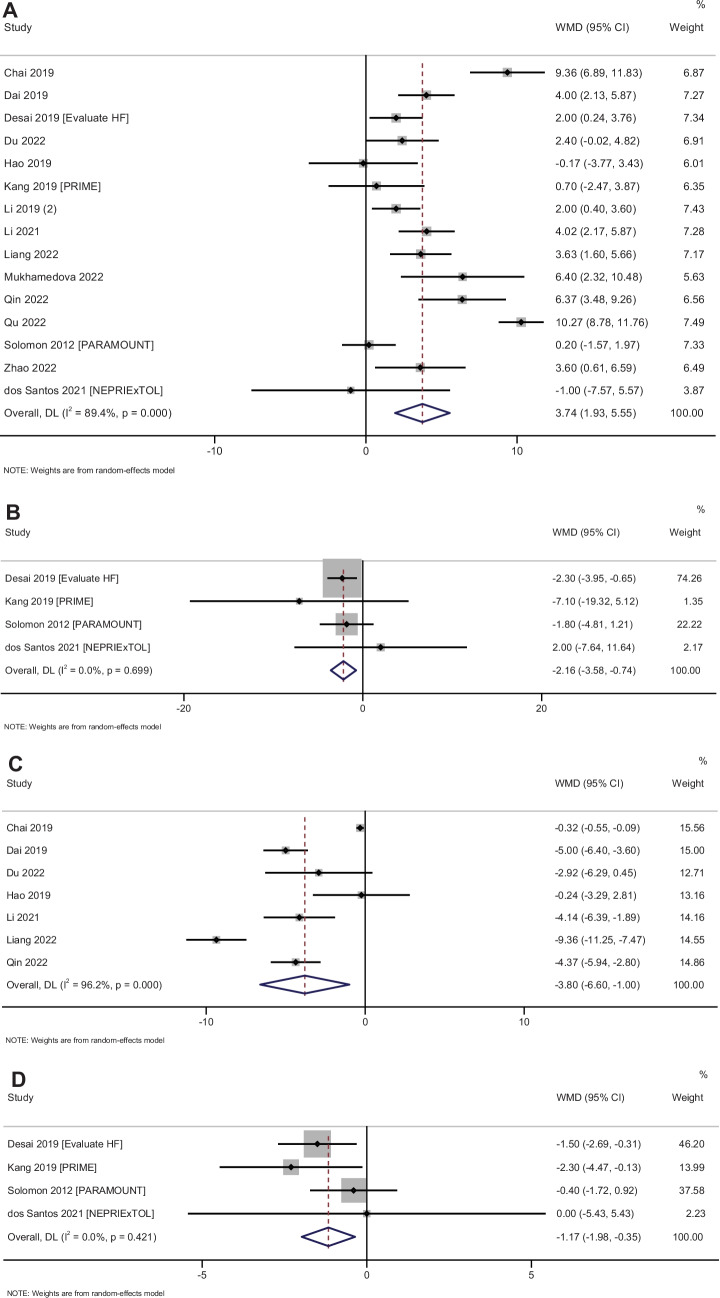
Forest plot showing the difference in echocardiographic parameters between sacubitril–valsartan and control group patients. **A** Left ventricular ejection fraction. **B** Left atrial volume index. **C** Left ventricular end-diastolic dimension. **D** *E*/*E*′ ratio

In total, 15 studies with 1994 participants have reported on the efficacy of sacubitril–valsartan on the LVEF amongst heart failure patients. The pooled MD was 3.74 (95% CI: 1.93 to 5.55; *I*^2^ = 89.4%), indicating that the patients receiving sacubitril–valsartan had significantly higher LVEF when compared to patients receiving any other medications (*p* < 0.001) (Fig. 5A).

Subgroup analysis based on the status of ejection fraction cannot be performed as all the studies reporting this outcome were conducted amongst reduced ejection fraction patients. Subgroup analysis based on the control group showed that the administration of sacubitril–valsartan was efficacious in improving LVEF irrespective of the type of control medications (ACE inhibitors/ARBs/placebos/conventional treatment) (Supplementary Fig. 12). Subgroup analysis based on the dose of sacubitril–valsartan showed that the 50 mg and 100 mg twice daily dosages showed significant improvement in LVEF, while studies with 200 mg twice daily dosage did not show statistical significance in the improvement of LVEF (Supplementary Fig. 13).

Assessment of publication bias revealed a symmetrical funnel plot with non-significant Egger’s test (*p* = 0.84), indicating the absence of publication bias (Supplementary Fig. 14). Univariable meta-regression was performed with variables such as country/study region, follow-up duration, dose of sacubitril–valsartan and type of control group. Amongst these variables, sacubitril–valsartan dose and control group had a *p* value less than 0.20 and it was included in the multivariable meta-regression model. The multivariable meta-regression model with these two variables was able to explain about 80% of the between-study variability.

#### LAVI

In total, 4 studies with 913 participants have reported on the efficacy of sacubitril–valsartan on the LAVI amongst heart failure patients. The pooled MD was −2.16 (95% CI: −3.58 to −0.74; *I*^2^ = 0%), indicating that the patients receiving sacubitril–valsartan had significantly lower LAVI when compared to patients in the control group (*p* = 0.003) (Fig. 5B). Subgroup analysis and publication bias assessment could not be performed due to limitation in the number of studies.

#### LVED

In total, 7 studies with 668 participants have reported on the efficacy of sacubitril–valsartan on the LVED amongst heart failure patients. The pooled MD was −3.80 (95% CI: −6.60 to −1.00; *I*^2^ = 96.8%), indicating that the patients receiving sacubitril–valsartan had significantly lower LVED when compared to patients in the control group (*p* = 0.008) (Fig. 5C). Subgroup analysis, meta-regression and publication bias assessment could not be performed due to limitation in the number of studies.

#### E/E′ ratio

In total, 4 studies with 913 participants have reported on the efficacy of sacubitril–valsartan on the *E*/*E*′ ratio amongst heart failure patients. The pooled MD was −1.16 (95% CI: −1.98 to −0.35; *I*^2^ = 96.8%), indicating that the patients receiving sacubitril–valsartan had a significantly lower *E*/*E*′ ratio when compared to patients in the control group (*p* = 0.005) (Fig. 5D). Subgroup analysis, meta-regression and publication bias assessment could not be performed due to limitation in the number of studies.

### Arrhythmia endpoints

#### Atrial arrhythmias

**Fig. 6:**
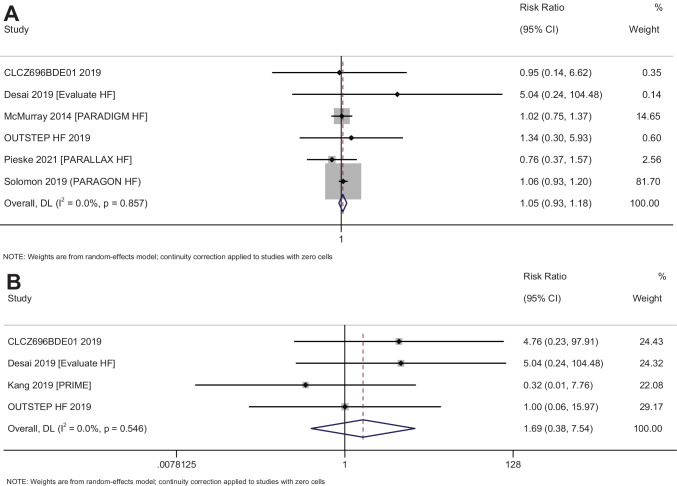
Forest plot showing the difference in arrhythmia endpoints between sacubitril–valsartan and control group patients. **A** Atrial arrhythmia. **B** Ventricular arrhythmia

In total, 6 studies with 17,053 participants have reported on the efficacy of sacubitril–valsartan on the atrial arrhythmias amongst heart failure patients. The pooled RR was 1.05 (95% CI: 0.93 to 1.17; *I*^2^ = 0%), indicating no significant difference between sacubitril–valsartan and control group patients in terms of atrial arrhythmias (*p* = 0.43) (Fig. 6A). Subgroup analysis and publication bias assessment could not be performed due to limitation in the number of studies.

#### Ventricular arrhythmias

In total, 4 studies with 1402 participants have reported on the efficacy of sacubitril–valsartan on the ventricular arrhythmias amongst heart failure patients. The pooled RR was 1.69 (95% CI: 0.38 to 7.54; *I*^2^ = 0%), indicating no significant difference between sacubitril–valsartan and control group patients in terms of ventricular arrhythmias (*p* = 0.49) (Fig. 6B). Subgroup analysis and publication bias assessment could not be performed due to limitation in the number of studies.

### Adverse events

#### Symptomatic hypotension

**Fig. 7:**
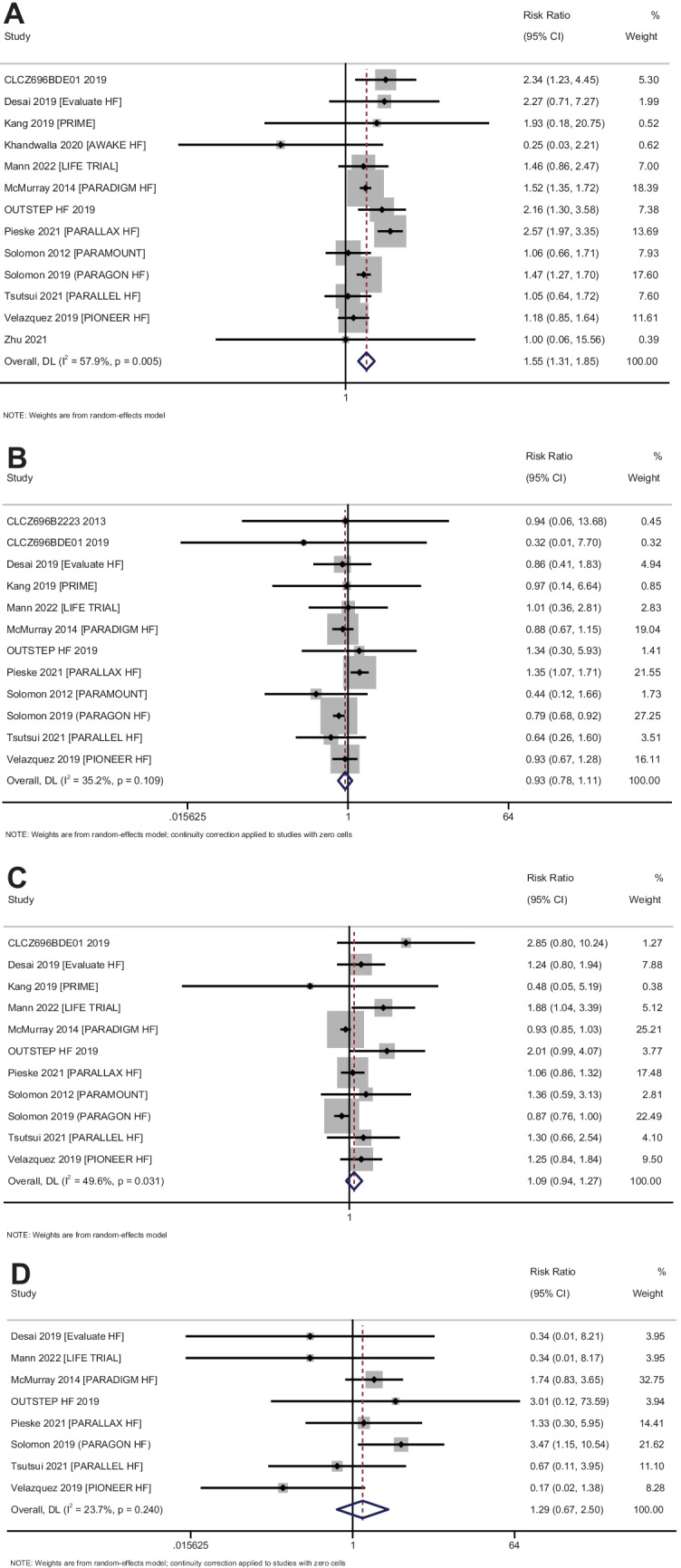
Forest plot showing the difference in adverse events between sacubitril–valsartan and control group patients. **A** Symptomatic hypotension. **B** Worsening renal function. **C** Hyperkalaemia. **D** Angioedema

In total, 13 studies with 19,150 participants have reported on the safety of sacubitril–valsartan against symptomatic hypotension amongst heart failure patients. The pooled RR was 1.55 (95% CI: 1.31 to 1.85; *I*^2^ = 57.9%), indicating that the patients receiving sacubitril–valsartan had a significantly higher risk of having symptomatic hypotension when compared to patients receiving any other medications (*p* < 0.001) (Fig. 7A).

Subgroup analysis based on the status of ejection fraction revealed that the patients with either reduced ejection fraction or preserved ejection fraction had a significantly higher risk of symptomatic hypotension following the administration of sacubitril–valsartan (Supplementary Fig. 15). Subgroup analysis based on the control group also showed that the administration of sacubitril–valsartan had a higher risk of symptomatic hypotension when compared to ACE inhibitors or ARBs (Supplementary Fig. 16). Subgroup analysis based on the dose of sacubitril–valsartan could not be performed as almost all the studies for this outcome used a dose of 200 mg twice daily. Assessment of publication bias revealed a symmetrical funnel plot with non-significant Egger’s test (*p* = 0.86), indicating the absence of publication bias (Supplementary Fig. 17).

#### Worsening of renal function

In total, 12 studies with 18,940 participants have reported on the safety of sacubitril–valsartan against worsening of renal function amongst heart failure patients. The pooled RR was 0.93 (95% CI: 0.78 to 1.11; *I*^2^ = 35.2%), indicating that the patients receiving sacubitril–valsartan did not have a significantly higher risk of having worsening of renal function when compared to patients receiving any other medications (*p* = 0.42) (Fig. 7B).

Subgroup analysis based on the status of ejection fraction revealed that the patients with either reduced ejection fraction or preserved ejection fraction did not have a higher risk of worsening of renal function following the administration of sacubitril–valsartan (Supplementary Fig. 18). Subgroup analysis based on the control group showed that the administration of sacubitril–valsartan had a significantly lower risk of worsening renal function (pooled RR = 0.79; 95% CI: 0.68–0.92) when compared to ARBs (Supplementary Fig. 19). Subgroup analysis based on the dose of sacubitril–valsartan could not be performed as almost all the studies for this outcome used a dose of 200 mg twice daily. Assessment of publication bias revealed a symmetrical funnel plot with non-significant Egger’s test (*p* = 0.87), indicating the absence of publication bias (Supplementary Fig. 20).

#### Hyperkalaemia

In total, 11 studies with 18,866 participants have reported on the safety of sacubitril–valsartan against hyperkalaemia amongst heart failure patients. The pooled RR was 1.09 (95% CI: 0.94 to 1.26; *I*^2^ = 49.6%), indicating that the patients receiving sacubitril–valsartan did not have a significantly higher risk of having hyperkalaemia when compared to patients receiving any other medications (*p* = 0.42) (Fig. 7C).

Subgroup analysis based on the status of ejection fraction revealed that the patients with either reduced ejection fraction or preserved ejection fraction did not have a higher risk of hyperkalaemia following the administration of sacubitril–valsartan (Supplementary Fig. 21). Subgroup analysis based on the control group also did not show any difference in the risk of hyperkalaemia depending on the type of control group (Supplementary Fig. 22). Subgroup analysis based on the dose of sacubitril–valsartan could not be performed as almost all the studies for this outcome used a dose of 200 mg twice daily. Assessment of publication bias revealed an asymmetrical funnel plot with significant Egger’s test (*p* = 0.007), indicating the presence of publication bias (Supplementary Fig. 23).

#### Angioedema

In total, 8 studies with 18,289 participants have reported on the safety of sacubitril–valsartan against angioedema amongst heart failure patients. The pooled RR was 1.29 (95% CI: 0.67 to 2.50; *I*^2^ = 23.7%), indicating that the patients receiving sacubitril–valsartan did not have a significantly higher risk of having angioedema when compared to patients receiving any other medications (*p* = 0.44) (Fig. 7D). Subgroup analysis and publication bias assessment could not be performed due to limitation in the number of studies.

### Blood parameter

#### NT-proBNP

**Fig. 8:**
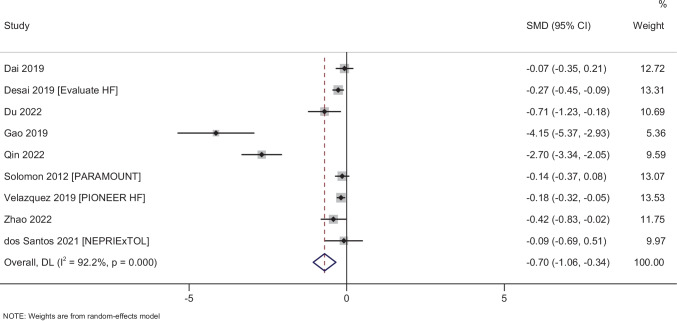
Forest plot showing the difference in NT-proBNP between sacubitril–valsartan and control group patients

In total, 9 studies with 2149 participants have reported on the efficacy of sacubitril–valsartan on the NT-proBNP amongst heart failure patients. The pooled MD was −0.70 (95% CI: −1.06 to −0.34; *I*^2^ = 92.2%), indicating that the patients receiving sacubitril–valsartan had significantly lower NT-proBNP when compared to patients in the control group (*p* < 0.001) (Fig. 8). Subgroup analysis, meta-regression and publication bias assessment could not be performed due to limitation in the number of studies.

#### Additional analysis

Sensitivity analysis has showed that there was no significant difference in the outcome in terms of magnitude of association or its direction for any of the above-mentioned outcomes.

## Discussion

The role of sacubitril–valsartan in the management of heart failure patients has been extensively studied, and it has been recommended to be an integral part of the management of these patients. Hence, it is important to study the efficacy and safety of sacubitril–valsartan across a wide range of parameters and multiple subgroups to provide a conclusive evidence and recommendations to clinical practice. Hence, this review was done to determine the efficacy and safety of sacubitril–valsartan on the management of heart failure patients.

In total, 34 studies were found to match the eligibility of the review, conducted mostly in China and having a higher risk of bias. We found that sacubitril–valsartan significantly reduces the adverse clinical outcomes such as all-cause mortality, cardiovascular mortality and hospitalizations for heart failure especially amongst patients with reduced ejection fraction. This was in line with the previous reviews reporting the efficacy of sacubitril–valsartan against mortality and hospitalization outcomes [15–18]. The distinguishing feature of our review is the comprehensive nature of the included studies (highest number of studies included across all these outcomes compared to previous reviews), subgroup analysis across multiple variables and additional analysis such as publication bias assessment and sensitivity analysis. Our review showed that the sacubitril–valsartan was more efficacious when compared to ACE inhibitors, while it was not significantly different compared to ARBs. However, the number of trials comparing these two medications was limited. Hence, more trials comparing ARNIs and ARBs are required to better understand the efficacy profile for heart failure patients.

Our review also showed favourable findings for sacubitril–valsartan with respect to echocardiographic findings such as LVEF, LAVI and LVED. This was also in line with the previous review reporting the difference in echocardiographic parameters between ARNIs and ACE inhibitors/ARBs [15]. The possible mechanism behind such protective effect of sacubitril–valsartan has been extensively reported in previous evidences. One of the commonest reported mechanisms is the simultaneous modulatory effects on calcium homeostasis and its role on major neurohormonal regulatory systems such as renin–angiotensin–aldosterone system (RAAS) and natriuretic peptide system (overactivated in heart failure patients) [56–58]. Improvement in the hemodynamic mechanism of heart failure patients by ARNIs can result in lesser oxidative stress and translational modification in intracellular ion channel involved in the calcium homeostasis leading to reduction in cardiac morbidity and mortality [56–59]. Additional mechanisms are related to the effect of ARNIs on natriuresis, decrease in wall stretch and myocardial fibrosis, vasodilation and reduction in sympathetic activation and inflammation [60–63].

There was no significant difference in terms of arrhythmia endpoints (both atrial and ventricular arrhythmias) between sacubitril–valsartan and control group patients. Previous studies assessing these endpoints have also reported no difference between these groups [64, 65]. Here also, the number of trials assessing these outcomes is limited and requires further large-scale trials to provide conclusive evidence on arrhythmia endpoints.

The safety profile of sacubitril–valsartan was also similar to the ACE inhibitors/ARBs/conventional treatment with respect to hyperkalaemia, worsening renal function and angioedema. However, only limitation with the sacubitril–valsartan was the higher risk of symptomatic hypotension when compared to other medications for heart failure. These findings were also in line with the previous reviews comparing the adverse events of ARNIs with control groups [17, 66]. We also found that sacubitril–valsartan significantly reduces the NT-proBNP when compared to ACE inhibitors/ARBs/placebo group medications amongst heart failure patients. All these findings show that the ARNIs have several protective efficacy parameters amongst heart failure patients.

### Strengths and limitations

This review has certain strengths. Only RCTs were included in this review, which improves the strength of evidence. Comprehensive search was conducted to reach the best possible evidence on this topic. No heterogeneity was found across almost all the outcomes, which might enhance the generalizability of the study findings. Sensitivity analysis also did not report any small study effects for any of the outcomes. Subgroup analysis was done across various important covariates, which might help in providing specific recommendations.

Despite these strengths, current meta-analysis has some limitations. Most included studies had a higher risk of bias, which might limit the credibility of the evidence. We found significant publication bias and heterogeneity across few outcomes. Hence, the study findings should be interpreted with caution. We tried to explore the source of heterogeneity using meta-regression across different variables. However, due to limitation of studies, it cannot be done for other outcomes reporting significant heterogeneity.

### Implications for clinicians and future research

Despite these limitations, this study has important implications for the clinicians and their practice. ARNIs have similar safety profile and better efficacy profile than ACE inhibitors/ARBs/any other forms of medications amongst heart failure patients. Hence, management of heart failure patients with ARNIs is important especially amongst the patients with reduced ejection fraction. This further promotes the longevity of the patients especially the chronic heart failure patients.

This review also supports the need for more RCTs on acute type of heart failure and on outcomes such as arrhythmia endpoints and quality of life. Future research should focus primarily on conducting a large-scale RCT, comparing multiple combined interventions and decide on the best possible intervention. Future RCTs should also strive towards disclosing conclusively the short-term and long-term effects of these medications to ensure proper management of heart failure patients.

## Supplementary Information

Below is the link to the electronic supplementary material.

Supplementary file1 (PDF 689 KB)

## Data Availability

Data will be made available upon reasonable request from researchers.

## References

[1] Groenewegen A, Rutten FH, Mosterd A, Hoes AW (2020). Epidemiology of heart failure. Eur J Heart Fail.

[2] Savarese G, Becher PM, Lund LH, Seferovic P, Rosano G, Coats AJ (2022) Global burden of heart failure: a comprehensive and updated review of epidemiology. Cardiovasc Res 10.1093/cvr/cvac013 35150240

[3] Shane N, Melissa B, Vivian M (2020). Extended-release oral milrinone for the treatment of heart failure with preserved ejection fraction. J Am Heart Assoc.

[4] Christiansen MN, Køber L, Torp-Pedersen C (2019). Prevalence of heart failure and other risk factors among first-degree relatives of women with peripartum cardiomyopathy. Heart.

[5] Anker MS, Hadzibegovic S, Lena A (2019). Recent advances in cardiooncology: a report from the ‘Heart Failure Association 2019 and World Congress on Acute Heart Failure 2019’. ESC Heart Fail.

[6] Legesse NY, Weldegebreal AS, Teklemariam DG (2020). Treatment optimization of beta-blockers in chronic heart failure therapy. Sci Rep.

[7] Kanan P, Fonarow Gregg C, Momanna A (2014). Calcium channel blockers and outcomes in older patients with heart failure and preserved ejection fraction. Circ Heart Fail.

[8] Kenji Y, Yuya M, Tetsuo Y (2019). Safety and prognostic impact of early treatment with angiotensin-converting enzyme inhibitors or angiotensin receptor blockers in patients with acute heart failure. Am J Cardiovasc Drugs.

[9] Pericas P, Mas-Lladó C, Ramis-Barceló MF (2021). Impact of sacubitril valsartan treatment on diastolic function in patients with heart failure and reduced ejection fraction. High Blood Press Cardiovasc Prev.

[10] Nicolas D, Kerndt CC, Reed M (2022) Sacubitril/valsartan. [Updated 2022 May 9]. In: StatPearls. Treasure Island (FL): StatPearls Publishing; 2022 Jan-. Available from: https://www.ncbi.nlm.nih.gov/books/NBK507904/

[11] Abelardo M-R, Mark RA, Burnett John C (2008). Biology of the natriuretic peptides. Am J Cardiol.

[12] Potter LR, Abbey-Hosch S, Dickey DM (2006). Natriuretic peptides, their receptors, and cyclic guanosine monophosphate-dependent signaling functions. Endocr Rev.

[13] Ponikowski P, Voors AA, Anker SD (2016). 2016 ESC guidelines for the diagnosis and treatment of acute and chronic heart failure: the task force for the diagnosis and treatment of acute and chronic heart failure of the European Society of cardiology (ESC)developed with the special contribution of the Heart Failure Association (HFA) of the ESC. Eur J Heart Fail.

[14] Yancy CW, Jessup M, Bozkurt B (2017). 2017 ACC/AHA/HFSA focused update of the 2013 ACCF/AHA guideline for the management of heart failure: a report of the American College of Cardiology/American Heart Association Task Force on Clinical Practice Guidelines and the Heart Failure Society of America. J Card Fail.

[15] Zheng C, Dai H, Huang J (2021). The efficacy and safety of sacubitril/valsartan in the treatment of chronic heart failure: a meta-analysis. Am J Transl Res.

[16] Lin J, Zhou J, Xie G, Liu J (2021) Efficacy and safety of sacubitril-valsartan in patients with heart failure: a systematic review and meta-analysis of randomized clinical trials: a PRISMA-compliant article. Medicine 100(52) 10.1097/MD.0000000000028231 PMC8718238 34967357

[17] Zhang H, Huang T, Shen W (2020). Efficacy and safety of sacubitril-valsartan in heart failure: a meta-analysis of randomized controlled trials. ESC Heart Failure.

[18] Yan Y, Liu B, Du J, Wang J, Jing X, Liu Y, Deng S, Du J, She Q (2021). SGLT2i versus ARNI in heart failure with reduced ejection fraction: a systematic review and meta-analysis. ESC Heart Failure.

[19] Page MJ, McKenzie JE, Bossuyt PM, Boutron I, Hoffmann TC, Mulrow CD, Shamseer L, Tetzlaff JM, Moher D (2021). Updating guidance for reporting systematic reviews: development of the PRISMA 2020 statement. J Clin Epidemiol.

[20] Sterne JA, Savović J, Page MJ, Elbers RG, Blencowe NS, Boutron I, Cates CJ, Cheng HY, Corbett MS, Eldridge SM, Emberson JR (2019). RoB 2: a revised tool for assessing risk of bias in randomised trials. BMJ.

[21] Higgins JP, Green S (2011) Cochrane handbook for systematic reviews of interventions. John Wiley & Sons

[22] Bano S, Bai P, Kumar S, Kumar N, Ali A, Pariya F (2021). Comparison of sacubitril/valsartan versus enalapril in the management of heart failure. Cureus.

[23] Du H, Li X, Zhao W, Jiang N (2022). The difference between sacubitril valsartan and valsartan on vascular endothelial function, APN, MMP-9, and BNP levels in patients with hypertension and chronic heart failure. M.A B, editor. J Healthc Eng.

[24] Huang SB, Chen H, Zhou H (2019). Curative effect observation of sacubitril valsartan in patients with heart failure with decreased cardiac ejection fraction. Neike.

[25] Kang DH, Park SJ, Shin SH, Hong GR, Lee S, Kim MS (2019). Angiotensin receptor neprilysin inhibitor for functional mitral regurgitation: PRIME study. Circulation.

[26] Khandwalla RM, Grant D, Birkeland K, Heywood JT, Fombu E, Owens RL (2021). The AWAKE-HF study: sacubitril/valsartan impact on daily physical activity and sleep in heart failure. Am J Cardiovasc Drugs.

[27] Li J, Cao J, Liu W et al (2019a) Efficacy of sacubitril/valsartan in the treatment of chronic heart failure in elderly patients with dilated cardiomyopathy. Zhonghua Laonianyixue Zazhi 38

[28] Li J, Cao J, Danzeng L (2019). Clinical effect of sacubitril valsartan sodium tablets on hypertensive patients with chronic heart failure in plateau area. Zhongguo Yiyao.

[29] Li BH, Fang KF, Lin PH, Zhang YH, Huang YX, Jie H (2021). Effect of sacubitril valsartan on cardiac function and endothelial function in patients with chronic heart failure with reduced ejection fraction. CH.

[30] Mann DL, Givertz MM, Vader JM, Starling RC, Shah P, McNulty SE (2022). Effect of treatment with sacubitril/valsartan in patients with advanced heart failure and reduced ejection fraction: a randomized clinical trial. JAMA Cardiol.

[31] McMurray JJV, Packer M, Desai AS, Gong J, Lefkowitz MP, Rizkala AR (2014). Angiotensin–neprilysin inhibition versus enalapril in heart failure. N Engl J Med.

[32] Qin J, Mo W, Xie L, Zhou E, Li G, Liang R (2022). Effect of sacubitril-valsartan combined with Zhenyuan capsule in the treatment of chronic heart failure comorbid anxiety and depression and its effect on inflammatory factors. NS.

[33] Solomon SD, Zile M, Pieske B, Voors A, Shah A, Kraigher-Krainer E (2012). The angiotensin receptor neprilysin inhibitor LCZ696 in heart failure with preserved ejection fraction: a phase 2 double-blind randomised controlled trial. The Lancet.

[34] Solomon SD, McMurray JJV, Anand IS, Ge J, Lam CSP, Maggioni AP (2019). Angiotensin–neprilysin inhibition in heart failure with preserved ejection fraction. N Engl J Med.

[35] Tsutsui H, Momomura S, ichi, Saito Y, Ito H, Yamamoto K, Sakata Y,  (2021). Efficacy and safety of sacubitril/valsartan in Japanese patients with chronic heart failure and reduced ejection fraction - results from the PARALLEL-HF study. Circ J.

[36] Velazquez EJ, Morrow DA, DeVore AD, Duffy CI, Ambrosy AP, McCague K (2019). Angiotensin–neprilysin inhibition in acute decompensated heart failure. N Engl J Med.

[37] Zhao Y, Tian L, Zhang L, Ma T, Di L, Wang Y (2022). The comparative effects of sacubitril/valsartan versus enalapril on pulmonary hypertension due to heart failure with a reduced ejection fraction. Pulm Circ.

[38] dos Santos MR, de Alves MJ, NN, Jordão CP, Pinto CEN, Correa KTS, de Souza FR,  (2021). Sacubitril/valsartan versus enalapril on exercise capacity in patients with heart failure with reduced ejection fraction: a randomized, double-blind, active-controlled study. Am Heart J.

[39] Wang G (2019). The effect of sacubitril–valsartan for the treatment of heart failure patients with mid-range ejection fraction. Strait Pharmaceutical J.

[40] Shi Y, Wang J, Han Y, Xu W, Song W, Gong Y (2020). Curative effect of sakubitril-valsartan on heart failure with preserved ejection fraction. Chin J Evid-Based Cardiovasc Med.

[41] Chen C, Jia B, Jiang S, Jiang S (2020). Clinical effect and prognosis of sacubitril-valsartan in treating heart failure patients with midrange ejection fraction. Chin J New Drugs Clin Rem.

[42] Chai DJ, Cheng K, Tu X (2019). The efficacy and safety of Entresto in treatment of patients with refractory heart failure. Xindian yu Xunhuan.

[43] Dai WL, Wu X, Wang S (2019). Clinical research on sacubitril/valsartan treating patients with chronic congestive heart failure. Zhongguo Yiyao.

[44] Desai AS, Solomon SD, Shah AM et al (2019) Effect of sacubitril-valsartan vs enalapril on aortic stiffness in patients with heart failure and reduced ejection fraction: a randomized clinical trial. JAMA 1077–10 10.1001/jama.2019.12843 PMC6749534 31475296

[45] Gao Y, Luan B, Gao Y (2019). Clinical observation of sacrubitril/valsartan in the treatment of chronic heart failure. Zhongguo Xun Zheng Xinxueguan Yixue Zazhi.

[46] Hao QM, Cheng J, Xue Y (2019). Comparison of the effects of sacubitril/valsartan and valsartan on heart and kidney function in patients with chronic heart failure. Xiandai Shengwuyixue Jinzhan.

[47] Tumasyan LL, Adamyan K, Chilingaryan A, Tunyan L, Mkrtchyan V (2019). Comparative efficacy of renin-angiotensin aldesteron system modulators and angiotensin receptor neprilyzin inhibitor in chronic heart failure with mid-ranged and preserved ejection fraction. Eur J Heart Fail.

[48] Novartis Pharmaceuticals (2019) Randomized study using accelerome try to compare sacubitril/valsartan and enalapril in patients with heart failure. Available: https://clinicaltrials.gov/ct2/show/nct02900378

[49] Novartis Pharmaceuticals (2013) Sodium excretion of LCZ696 in patients with hypertension; heart failure and healthy volunteers. Available: https://clinicaltrials.gov/ct2/show/NCT01353508

[50] Novartis Pharmaceuticals (2019) Exercise capacity study of LCZ696 vs. enalapril in patients with chronic heart failure and reduced ejection fraction. Available: https://clinicaltrials.gov/ct2/show/NCT02768298

[51] Pieske B, Wachter R, Shah SJ (2021). Effect of sacubitril/valsartan vs standard medical therapies on plasma NT-proBNP concentration and submaximal exercise capacity in patients with heart failure and preserved ejection fraction: the PARALLAX randomized clinical trial. JAMA.

[52] Qu XH (2022). Analysis of the clinical effect of sacubitril valsartan sodium and benazepril in the treatment of chronic heart failure. Chinese Journal of Modern Drug Applications.

[53] Liang WH (2022). The effect of sacubitril and valsartan in the treatment of elderly patients with heart failure with reduced ejection fraction and its influence on cardiac function. Chinese Journal of Modern Drug Applications.

[54] Zhu T, Zheng G, Sheng X (2021). Comparison of efficacy and safety of sacubitril valsartan sodium tablets and benazepril hydrochloride tablets in the treatment of chronic heart failure with reduced ejection fraction. Evaluation and Analysis of Drug-Use in Hospitals of China.

[55] Mukhamedova M, Narzullaeva DS, Uzokov JK (2022) Influence of sacubitril/valsartan on hibernating myocardium in patients with chronic heart failure with reduced ejection fraction. Eur J Cardiovasc Nurs 21(1):zvac060.007. 10.1093/eurjcn/zvac060.007

[56] Alvarez CK, Cronin E, Baker WL, Kluger J (2019). Heart failure as a substrate and trigger for ventricular tachycardia. J Interv Card Electrophysiol.

[57] D’Elia E, Iacovoni A, Vaduganathan M, Lorini FL, Perlini S, Senni M (2017). Neprilysin inhibition in heart failure: mechanisms and substrates beyond modulating natriuretic peptides. Eur J Heart Fail.

[58] Packer M (2019). Neurohormonal antagonists are preferred to an implantable cardioverter-defibrillator in preventing sudden death in heart failure. JACC Heart Fail.

[59] Dridi H, Kushnir A, Zalk R, Yuan Q, Melville Z, Marks AR (2020). Intracellular calcium leak in heart failure and atrial fibrillation: a unifying mechanism and therapeutic target. Nat Rev Cardiol.

[60] Valentim Gonçalves A, Pereira-da-Silva T, Galrinho A (2019). Antiarrhythmic effect of sacubitril-valsartan: cause or consequence of clinical improvement?. J Clin Med.

[61] Dargad RR, Prajapati MR, Dargad RR, Parekh JD (2018). Sacubitril/valsartan: a novel angiotensin receptor-neprilysin inhibitor. Indian Heart.

[62] Sarrias A, Bayes-Genis A (2018). Is sacubitril/valsartan (also) an antiarrhythmic drug?. Circulation.

[63] Pfau D, Thorn SL, Zhang J (2019). Angiotensin receptor neprilysin inhibitor attenuates myocardial remodeling and improves infarct perfusion in experimental heart failure. Sci Rep.

[64] Liu X, Liu H, Wang L, Zhang L, Xu Q (2022). Role of sacubitril-valsartan in the prevention of atrial fibrillation occurrence in patients with heart failure: a systematic review and meta-analysis of randomized controlled trials. PLoS ONE.

[65] Fernandes ADF, Fernandes GC, Ternes CMP, Cardoso R, Chaparro SV, Goldberger JJ (2021) Sacubitril/valsartan versus angiotensin inhibitors and arrhythmia endpoints in heart failure with reduced ejection fraction. Heart Rhythm O2. 2(6Part B):724–732. 10.1016/j.hroo.2021.09.009 10.1016/j.hroo.2021.09.009 PMC8710618 34988523

[66] Charuel E, Menini T, Bedhomme S, Pereira B, Piñol-Domenech N, Bouchant S, Boussageon R, Bœuf-Gibot S, Vaillant-Roussel H (2021). Benefits and adverse effects of sacubitril/valsartan in patients with chronic heart failure: a systematic review and meta-analysis. Pharmacol Res Perspect.

