# Supplementary material for: Efficacy and safety profile of angiotensin receptor neprilysin inhibitors in the management of heart failure: a systematic review and meta-analysis of randomized controlled trials
Source: Heart Fail Rev. 2022 Oct 3;28(4):905–23. doi: 10.1007/s10741-022-10273-3 (PMC10289972; doi:10.1007/s10741-022-10273-3)

Supplementary text 1: Search strategy

Supplementary Figure 1: Forest plot showing difference in all-cause mortality between sacubitril-valsartan and control group based on status of ejection fraction

Supplementary Figure 2: Forest plot showing difference in all-cause mortality between sacubitril-valsartan and control group based on type of comparison group

Supplementary Figure 3: Funnel plot for all-cause mortality outcome

Supplementary Figure 4: Forest plot showing difference in cardiovascular mortality between sacubitril-valsartan and control group based on status of ejection fraction

Supplementary Figure 5: Forest plot showing difference in cardiovascular mortality between sacubitril-valsartan and control group based on type of comparison group

Supplementary Figure 6: Funnel plot for cardiovascular mortality outcome

Supplementary Figure 7: Forest plot showing difference in hospitalization between sacubitril-valsartan and control group based on status of ejection fraction

Supplementary Figure 8: Forest plot showing difference in hospitalization between sacubitril-valsartan and control group based on type of comparison group

Supplementary Figure 9: Funnel plot for hospitalization outcome

Supplementary Figure 10: Forest plot showing difference in quality of life between sacubitril-valsartan and control group

Supplementary Figure 11: Forest plot showing difference in improvement of NYHA functional status between sacubitril-valsartan and control group

Supplementary Figure 12: Forest plot showing difference in left ventricular ejection fraction between sacubitril-valsartan and control group based on type of comparison group

Supplementary Figure 13: Forest plot showing difference in left ventricular ejection fraction between sacubitril-valsartan and control group based on dose of sacubitril-valsartan

Supplementary Figure 14: Funnel plot for left ventricular ejection fraction

Supplementary Figure 15: Forest plot showing difference in symptomatic hypotension between sacubitril-valsartan and control group based on status of ejection fraction

Supplementary Figure 16: Forest plot showing difference in symptomatic hypotension between sacubitril-valsartan and control group based on type of comparison group

Supplementary Figure 17: Funnel plot for symptomatic hypotension outcome

Supplementary Figure 18: Forest plot showing difference in worsening renal function between sacubitril-valsartan and control group based on status of ejection fraction

Supplementary Figure 19: Forest plot showing difference in worsening renal function between sacubitril-valsartan and control group based on type of comparison group

Supplementary Figure 20: Funnel plot for worsening renal function outcome

Supplementary Figure 21: Forest plot showing difference in hyperkalaemia between sacubitril-valsartan and control group based on status of ejection fraction

Supplementary Figure 22: Forest plot showing difference in hyperkalaemia between sacubitril-valsartan and control group based on type of comparison group

Supplementary Figure 23: Funnel plot for hyperkalaemia outcome

Supplementary text 1: Search strategy:

Strategy 1: (("receptors, angiotensin"[MeSH Terms] OR ("receptors"[All Fields] AND "angiotensin"[All Fields]) OR "angiotensin receptors"[All Fields] OR ("angiotensin"[All Fields] AND "receptor"[All Fields]) OR "angiotensin receptor"[All Fields]) AND ("neprilysin"[MeSH Terms] OR "neprilysin"[All Fields] OR "neprilysins"[All Fields]) AND ("antagonists and inhibitors"[MeSH Subheading] OR ("antagonists"[All Fields] AND "inhibitors"[All Fields]) OR "antagonists and inhibitors"[All Fields] OR "inhibitors"[All Fields] OR "inhibitor"[All Fields] OR "inhibitor s"[All Fields]) AND ("heart failure"[MeSH Terms] OR ("heart"[All Fields] AND "failure"[All Fields]) OR "heart failure"[All Fields])) AND (randomizedcontrolledtrial[Filter])

Translations

Angiotensin Receptor: "receptors, angiotensin"[MeSH Terms] OR ("receptors"[All Fields] AND "angiotensin"[All Fields]) OR "angiotensin receptors"[All Fields] OR ("angiotensin"[All Fields] AND "receptor"[All Fields]) OR "angiotensin receptor"[All Fields]

Neprilysin: "neprilysin"[MeSH Terms] OR "neprilysin"[All Fields] OR "neprilysins"[All Fields]

Inhibitors: "antagonists and inhibitors"[Subheading] OR ("antagonists"[All Fields] AND "inhibitors"[All Fields]) OR "antagonists and inhibitors"[All Fields] OR "inhibitors"[All Fields] OR "inhibitor"[All Fields] OR "inhibitor's"[All Fields]

heart failure: "heart failure"[MeSH Terms] OR ("heart"[All Fields] AND "failure"[All Fields]) OR "heart failure"[All Fields] – 93 results

Strategy 2: (("sacubitril and valsartan sodium hydrate drug combination"[Supplementary Concept] OR "sacubitril and valsartan sodium hydrate drug combination"[All Fields] OR "sacubitril valsartan"[All Fields]) AND ("heart failure"[MeSH Terms] OR ("heart"[All Fields] AND "failure"[All Fields]) OR "heart failure"[All Fields])) AND (randomizedcontrolledtrial[Filter])

Translations

sacubitril-valsartan: "sacubitril and valsartan sodium hydrate drug combination"[Supplementary Concept] OR "sacubitril and valsartan sodium hydrate drug combination"[All Fields] OR "sacubitril valsartan"[All Fields]

heart failure: "heart failure"[MeSH Terms] OR ("heart"[All Fields] AND "failure"[All Fields]) OR "heart failure"[All Fields] – 134 results

Supplementary Figure 1: Forest plot showing difference in all-cause mortality between sacubitril-valsartan and control group based on status of ejection fraction

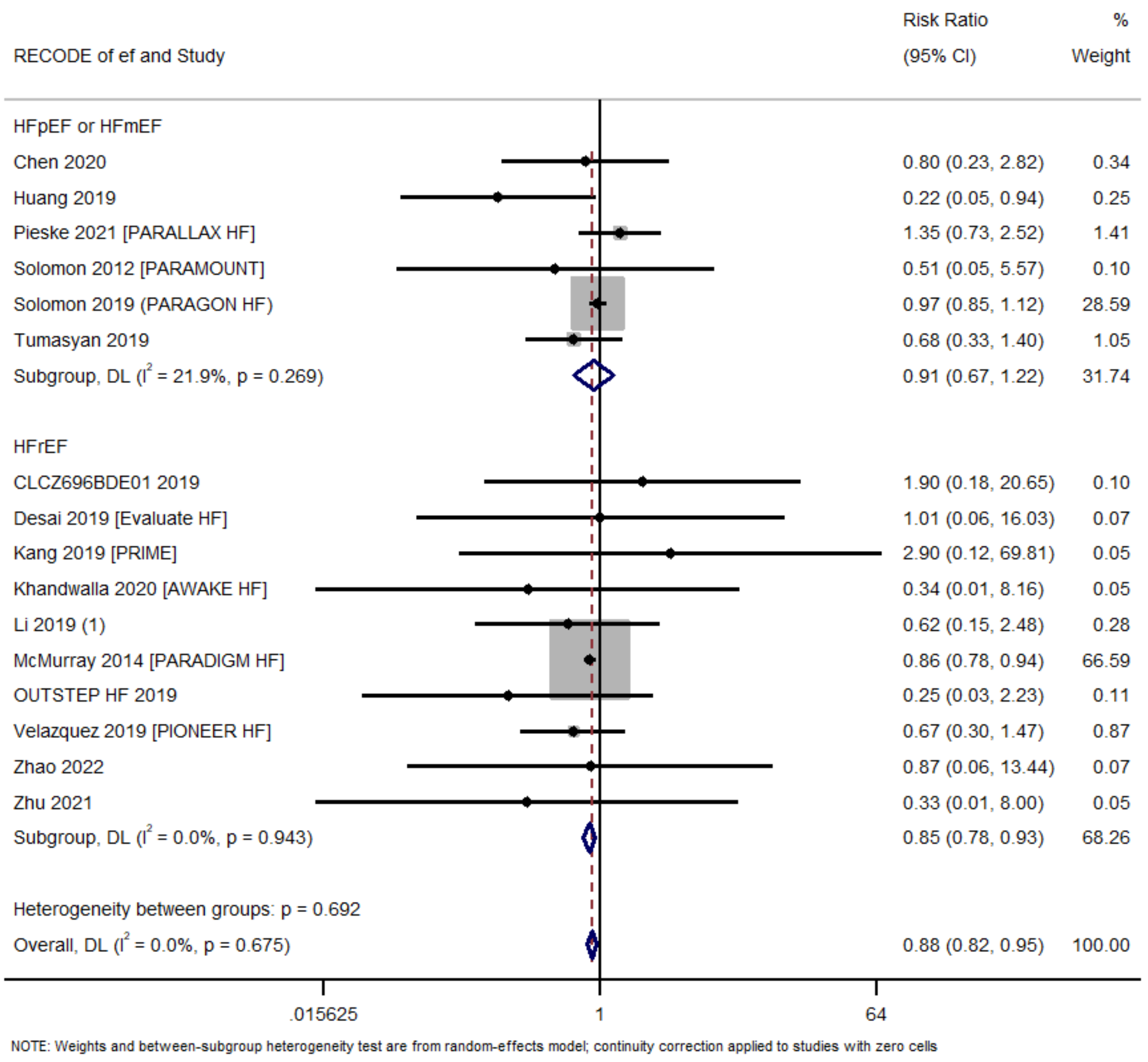

Supplementary Figure 2: Forest plot showing difference in all-cause mortality between sacubitril-valsartan and control group based on type of comparison group

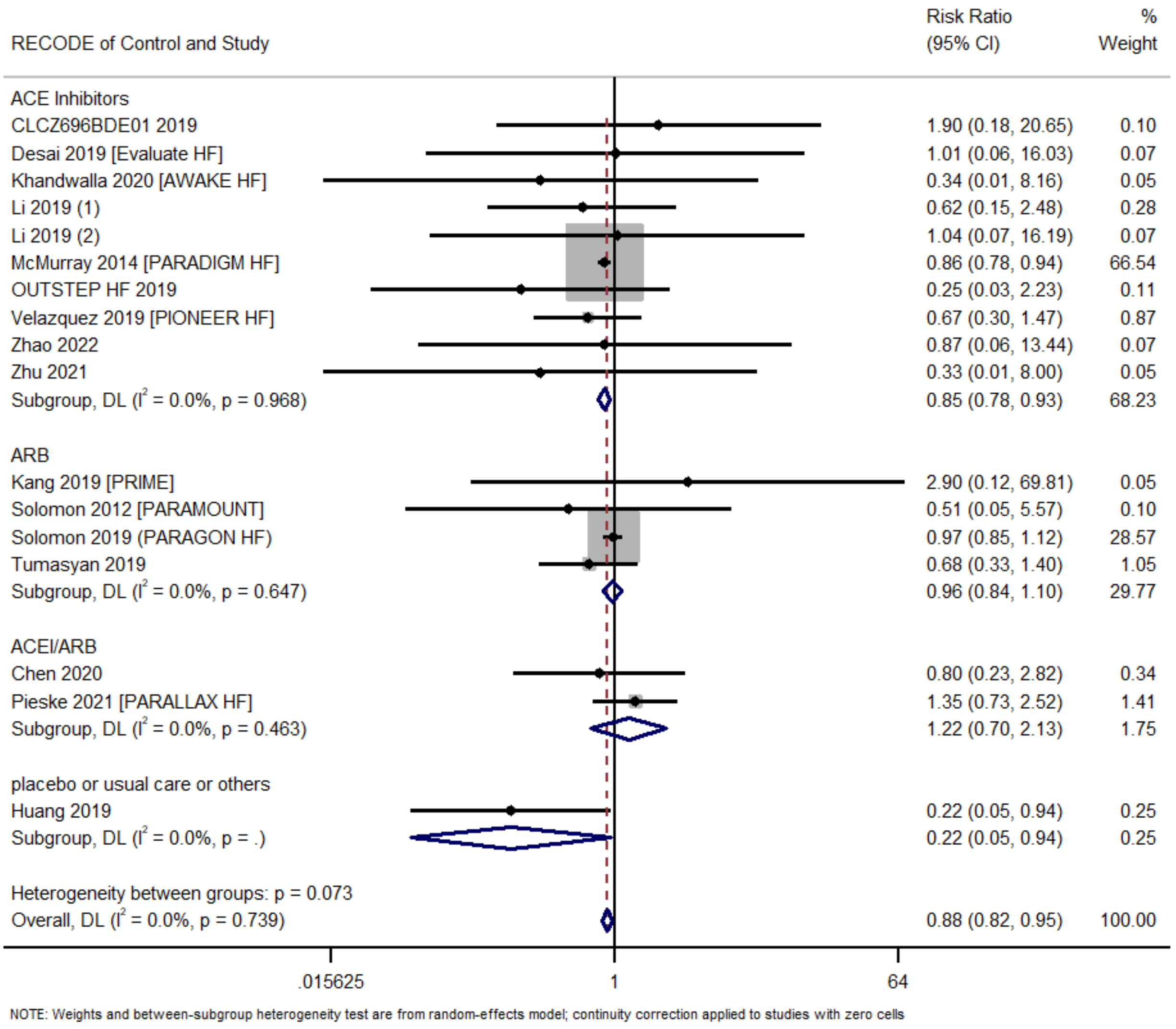

Supplementary Figure 3: Funnel plot for all-cause mortality outcome

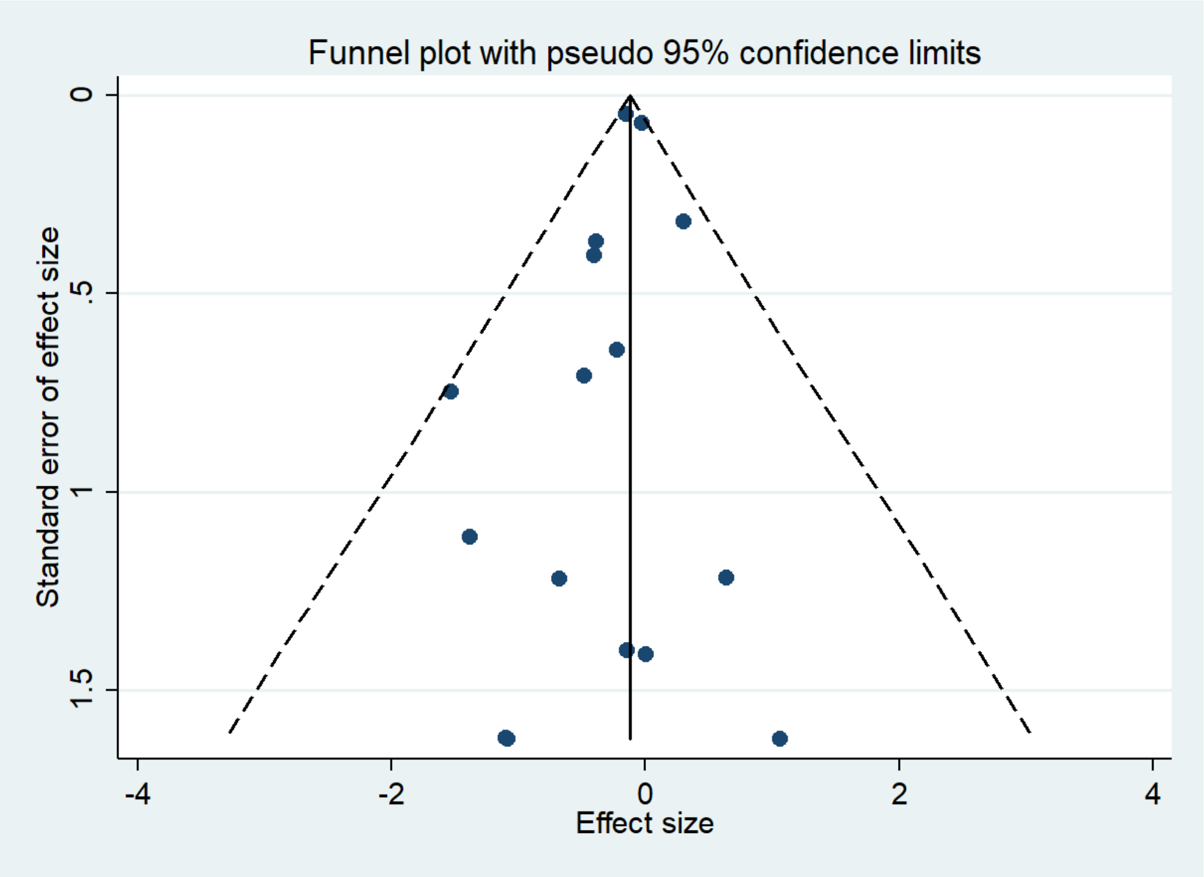

Supplementary Figure 4: Forest plot showing difference in cardiovascular mortality between sacubitril-valsartan and control group based on status of ejection fraction

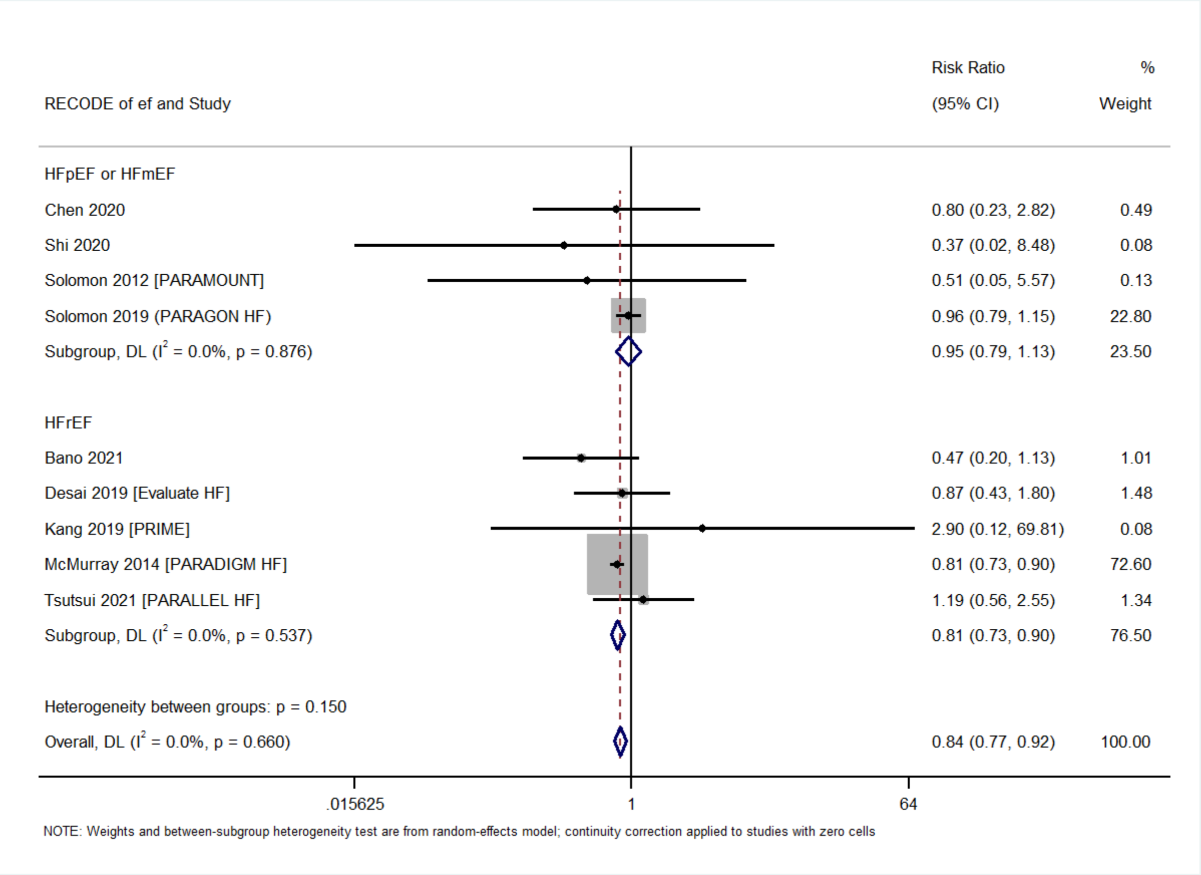

Supplementary Figure 5: Forest plot showing difference in cardiovascular mortality between sacubitril-valsartan and control group based on type of comparison group

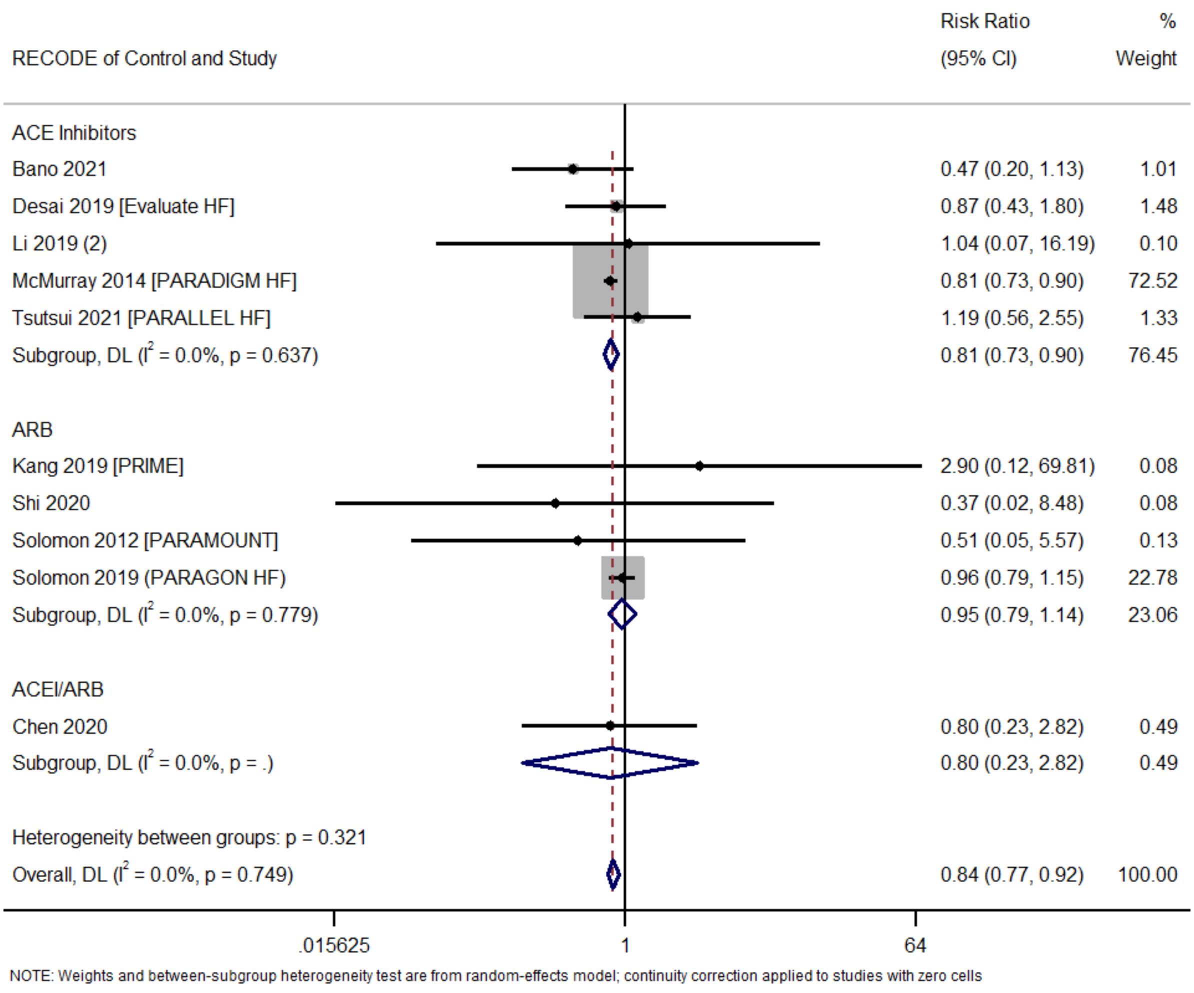

Supplementary Figure 6: Funnel plot for cardiovascular mortality outcome

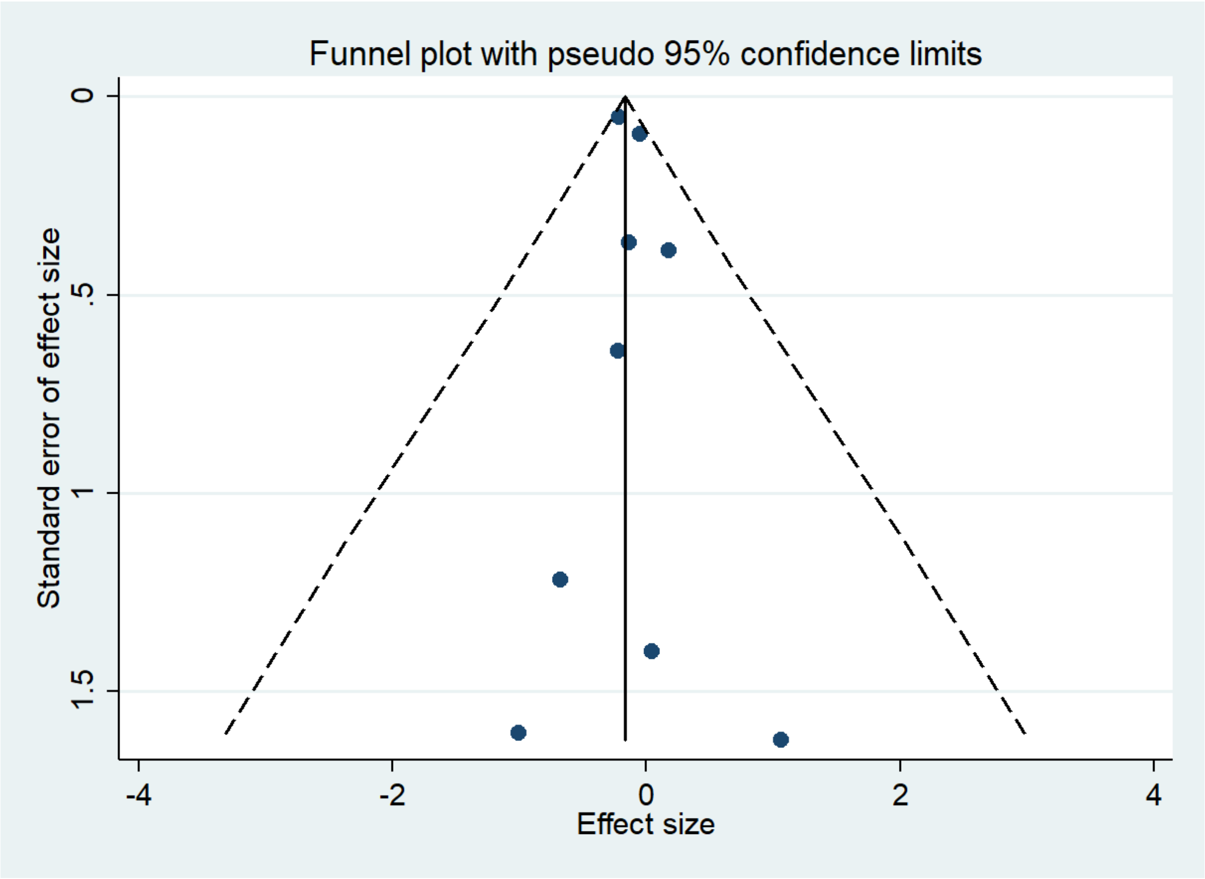

Supplementary Figure 7: Forest plot showing difference in hospitalization between sacubitril-valsartan and control group based on status of ejection fraction

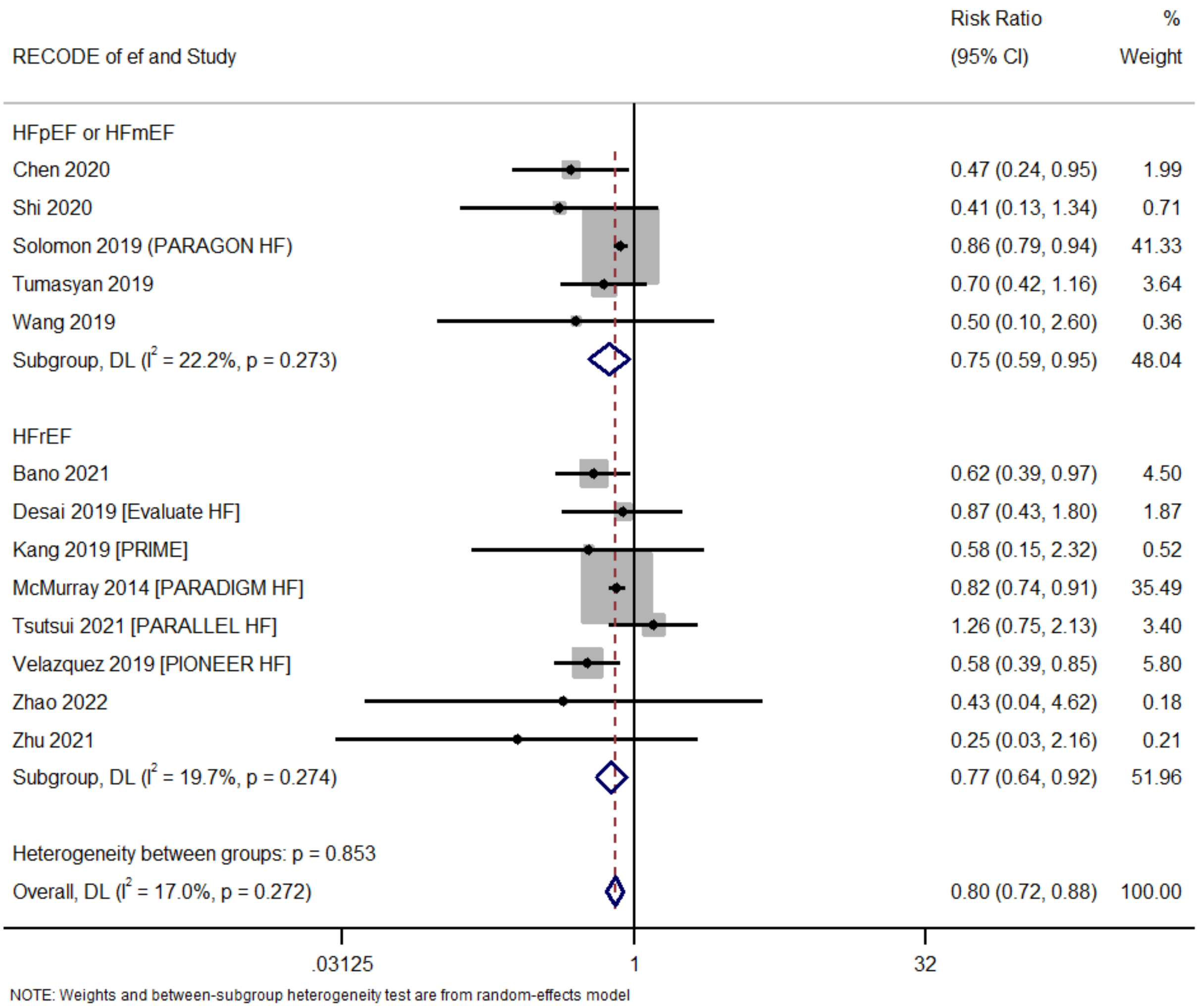

Supplementary Figure 8: Forest plot showing difference in hospitalization between sacubitril-valsartan and control group based on type of comparison group

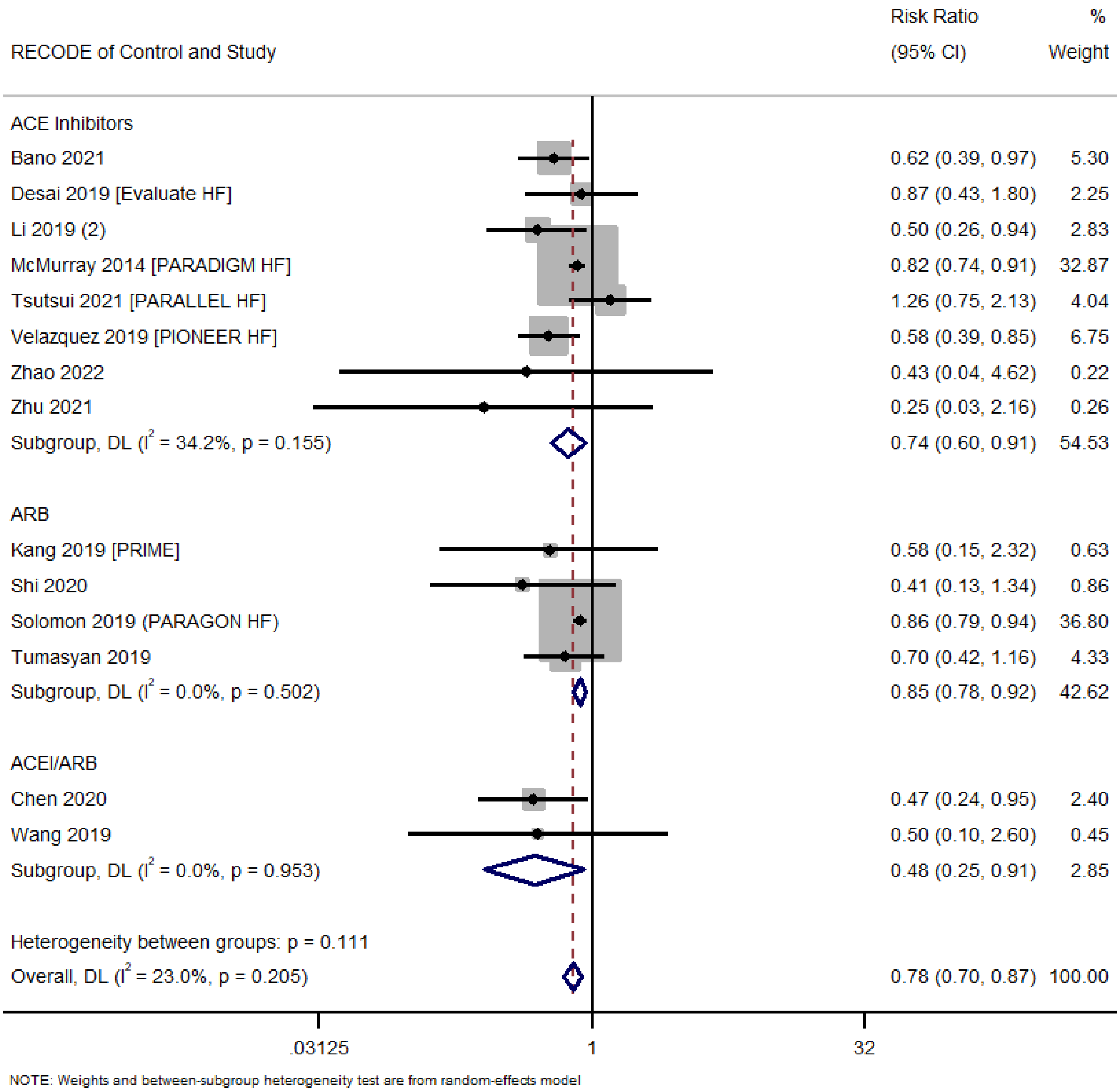

Supplementary Figure 9: Funnel plot for hospitalization outcome

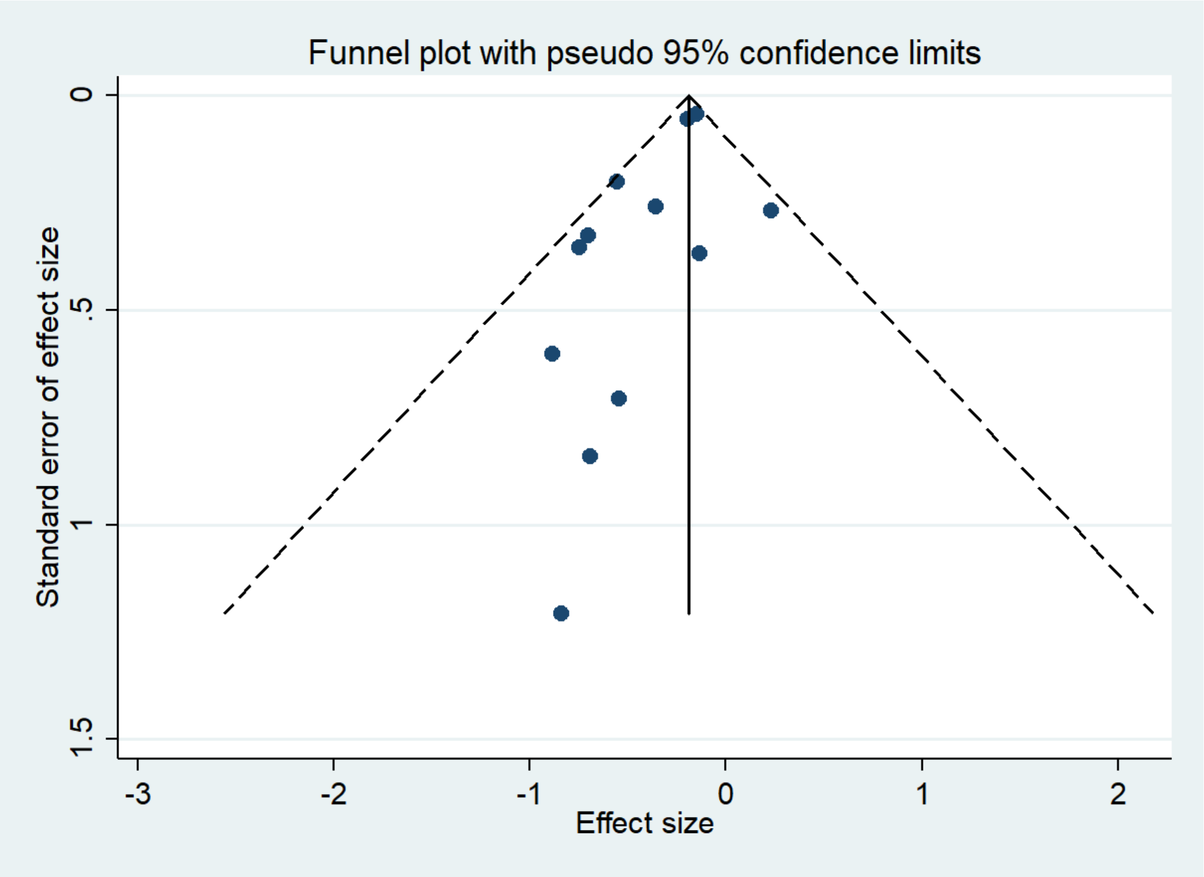

Supplementary Figure 10: Forest plot showing difference in quality of life between sacubitril-valsartan and control group

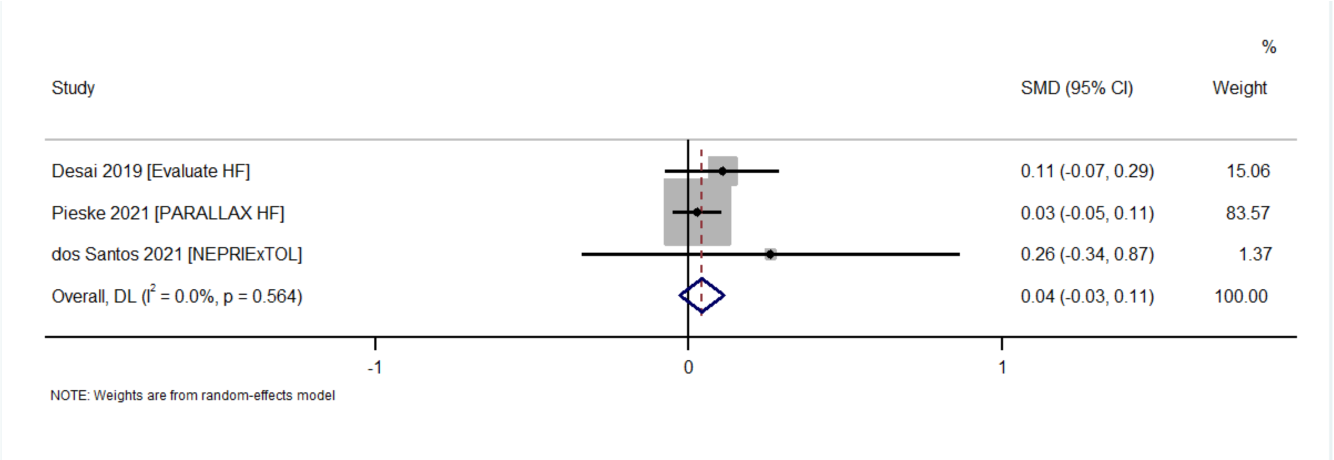

Supplementary Figure 11: Forest plot showing difference in improvement of NYHA functional status between sacubitril-valsartan and control group

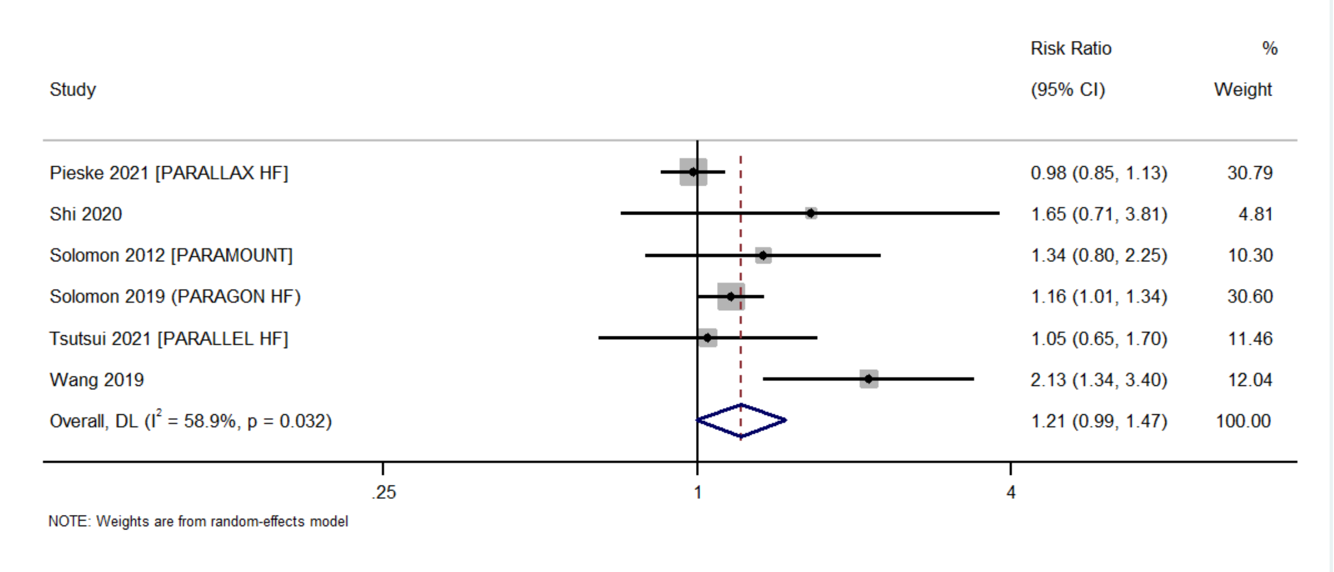

Supplementary Figure 12: Forest plot showing difference in left ventricular ejection fraction between sacubitril-valsartan and control group based on type of comparison group

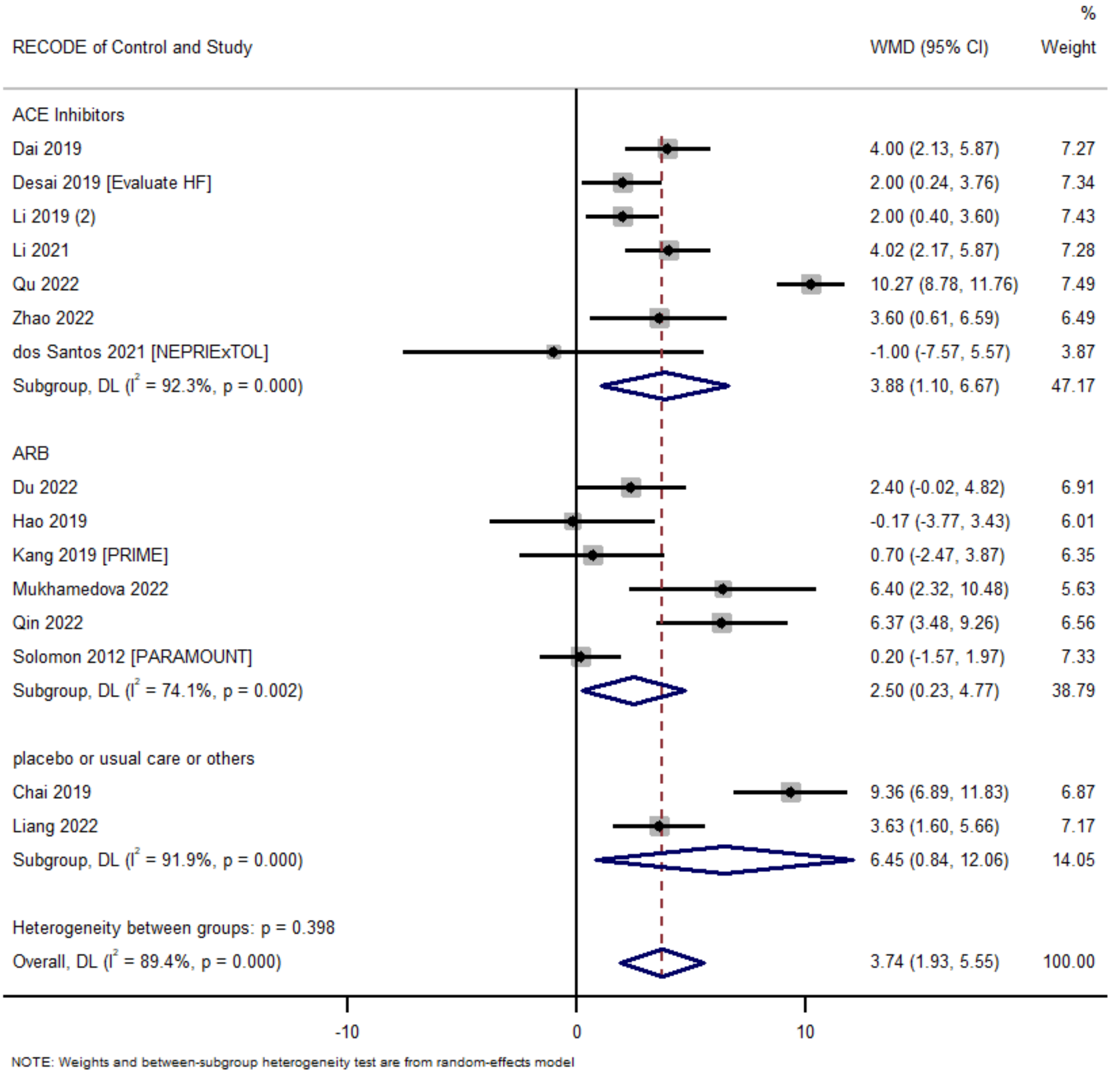

Supplementary Figure 13: Forest plot showing difference in left ventricular ejection fraction between sacubitril-valsartan and control group based on dose of sacubitril-valsartan

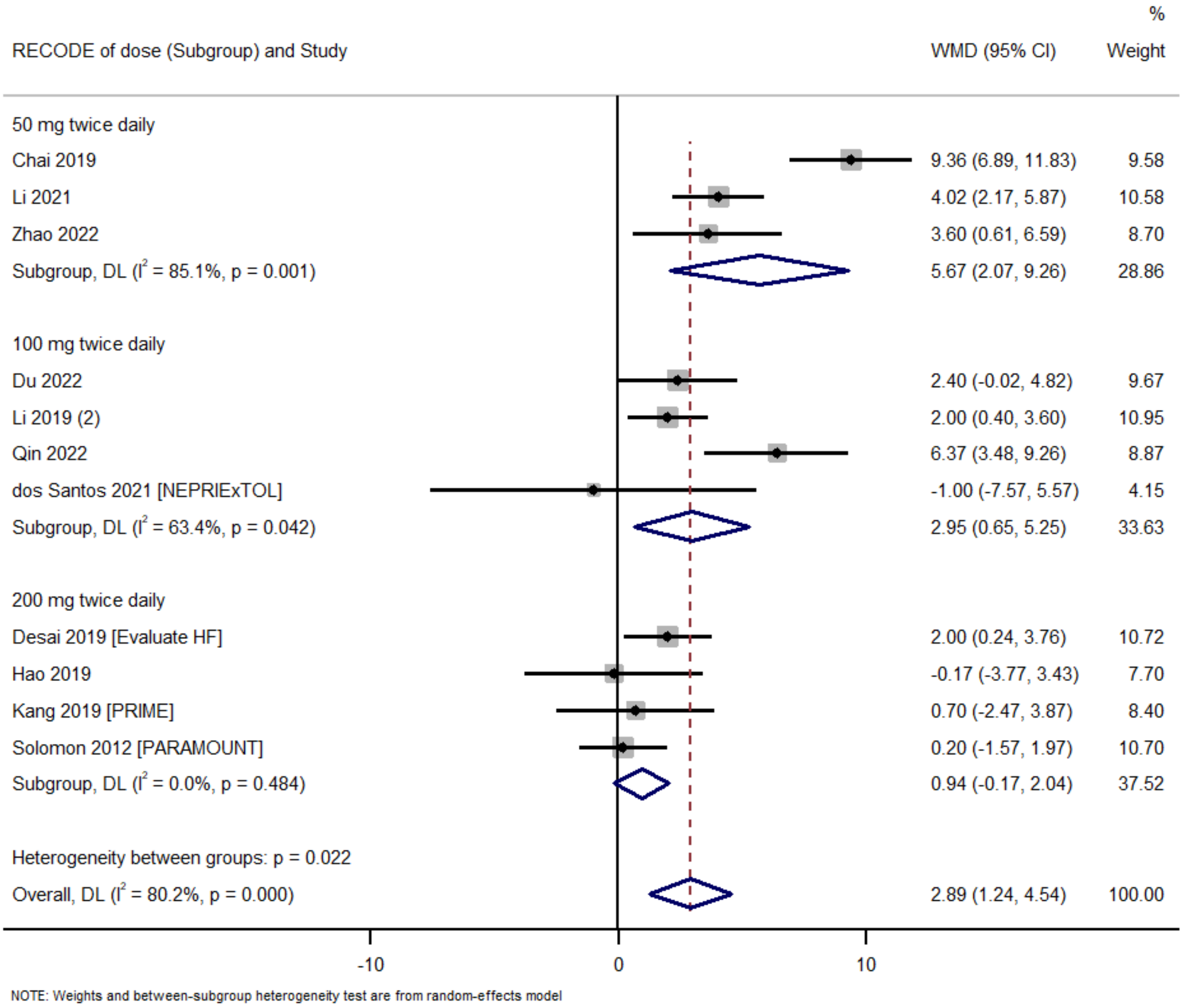

Supplementary Figure 14: Funnel plot for left ventricular ejection fraction

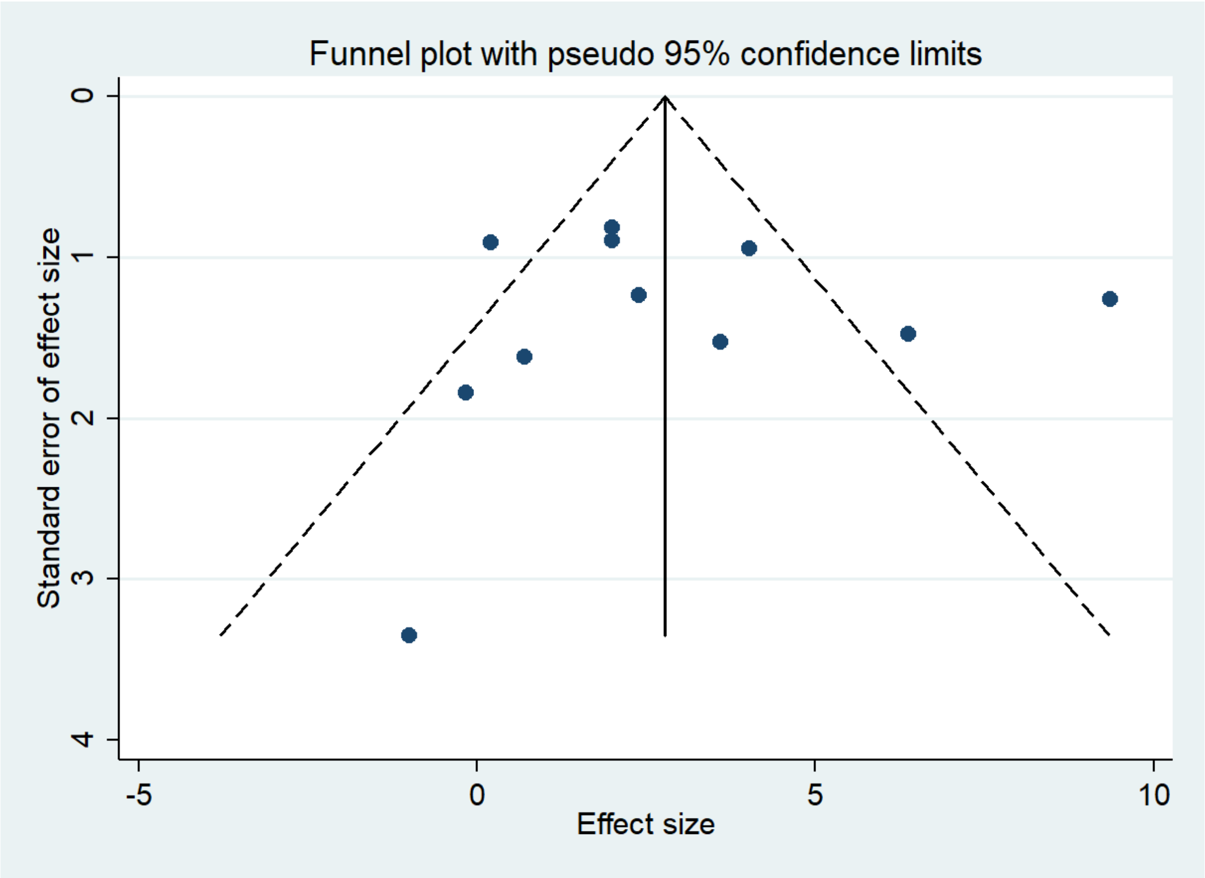

Supplementary Figure 15: Forest plot showing difference in symptomatic hypotension between sacubitril-valsartan and control group based on status of ejection fraction

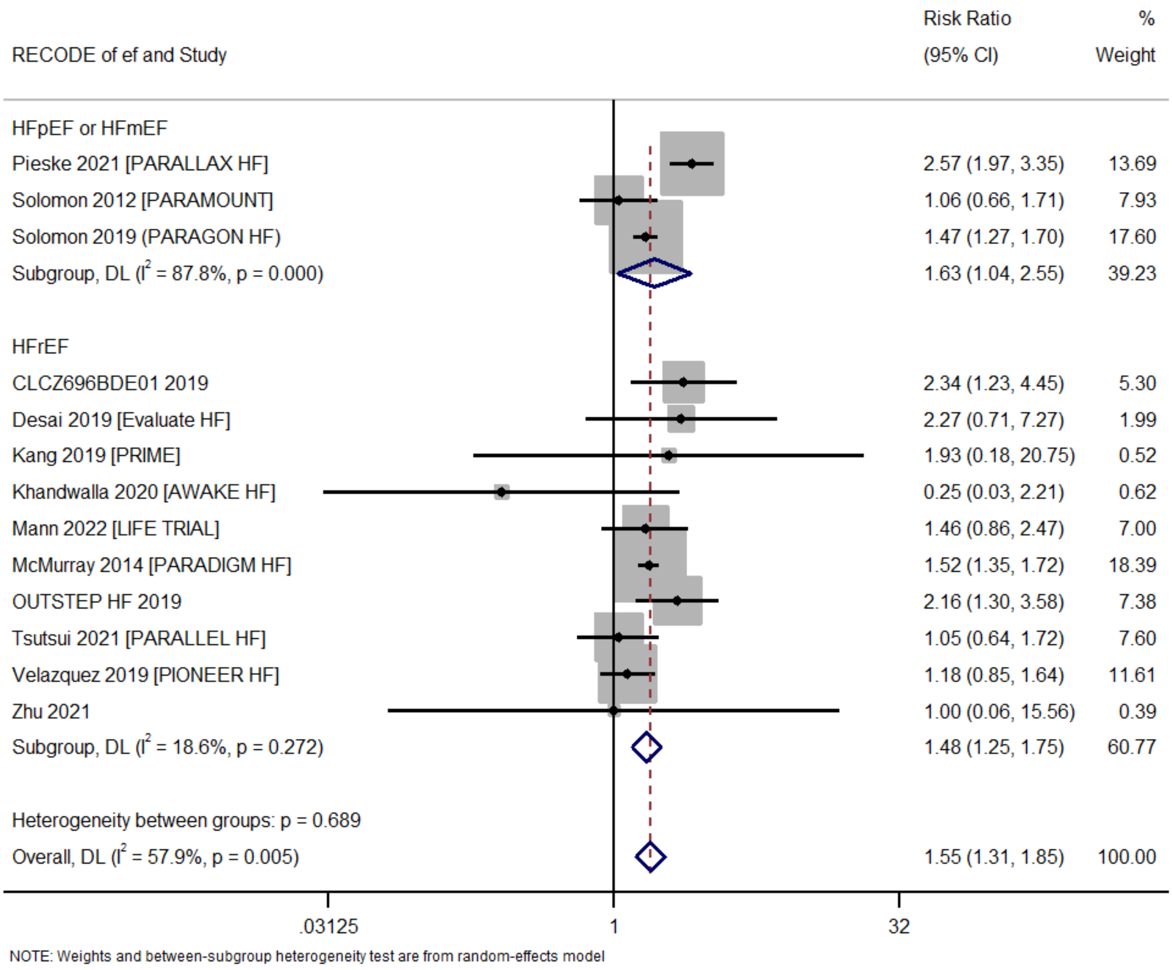

Supplementary Figure 16: Forest plot showing difference in symptomatic hypotension between sacubitril-valsartan and control group based on type of comparison group

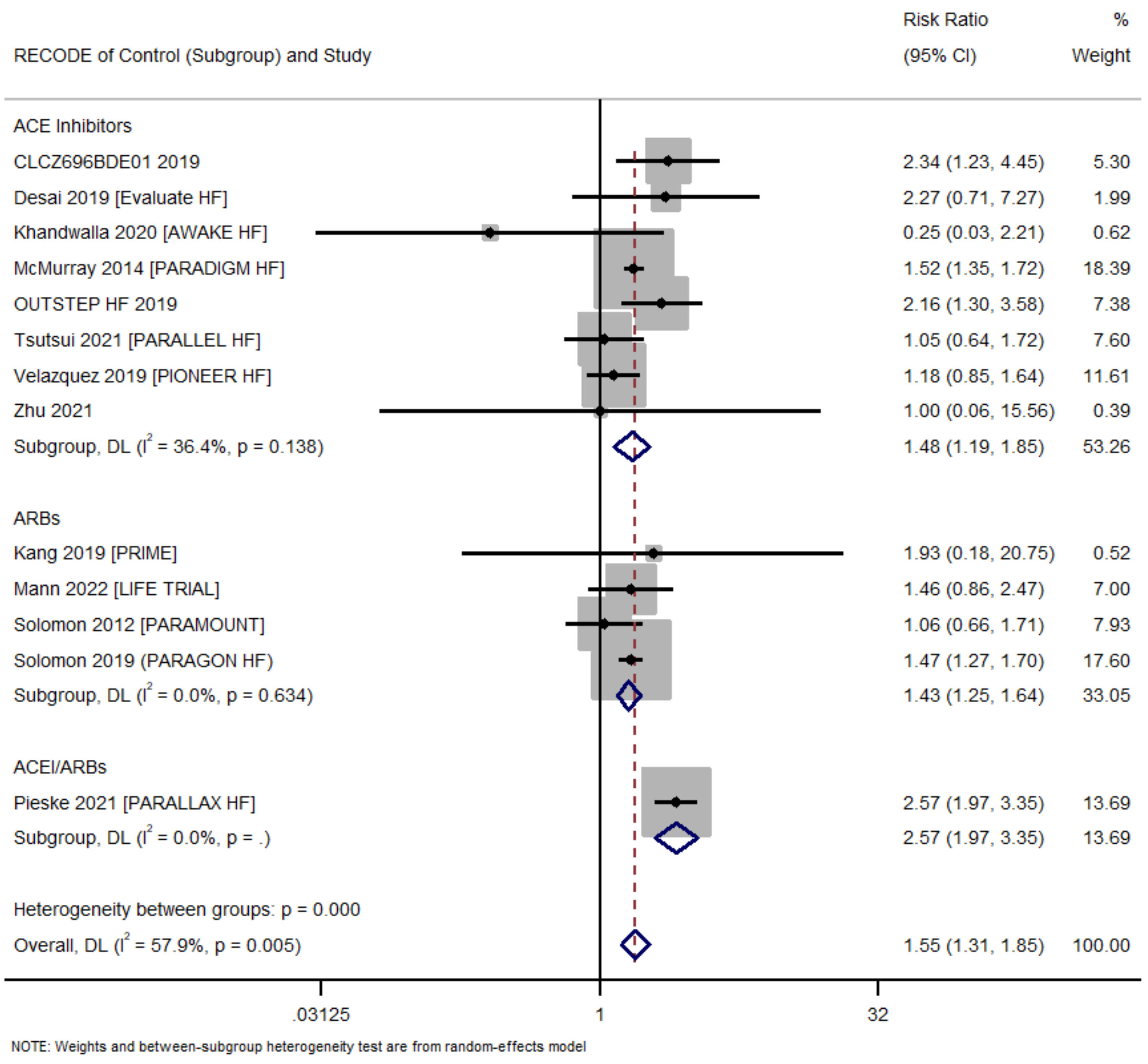

Supplementary Figure 17: Funnel plot for symptomatic hypotension outcome

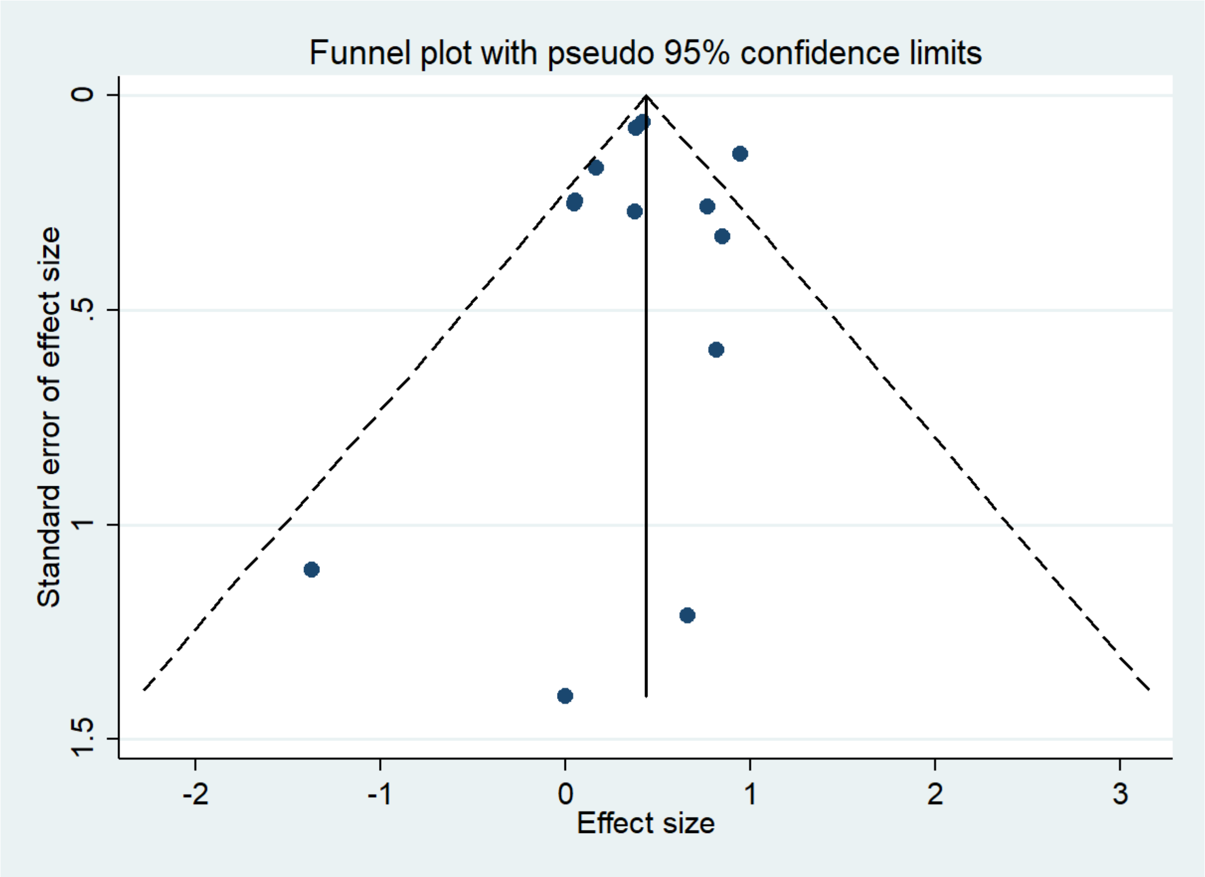

Supplementary Figure 18: Forest plot showing difference in worsening renal function between sacubitril-valsartan and control group based on status of ejection fraction

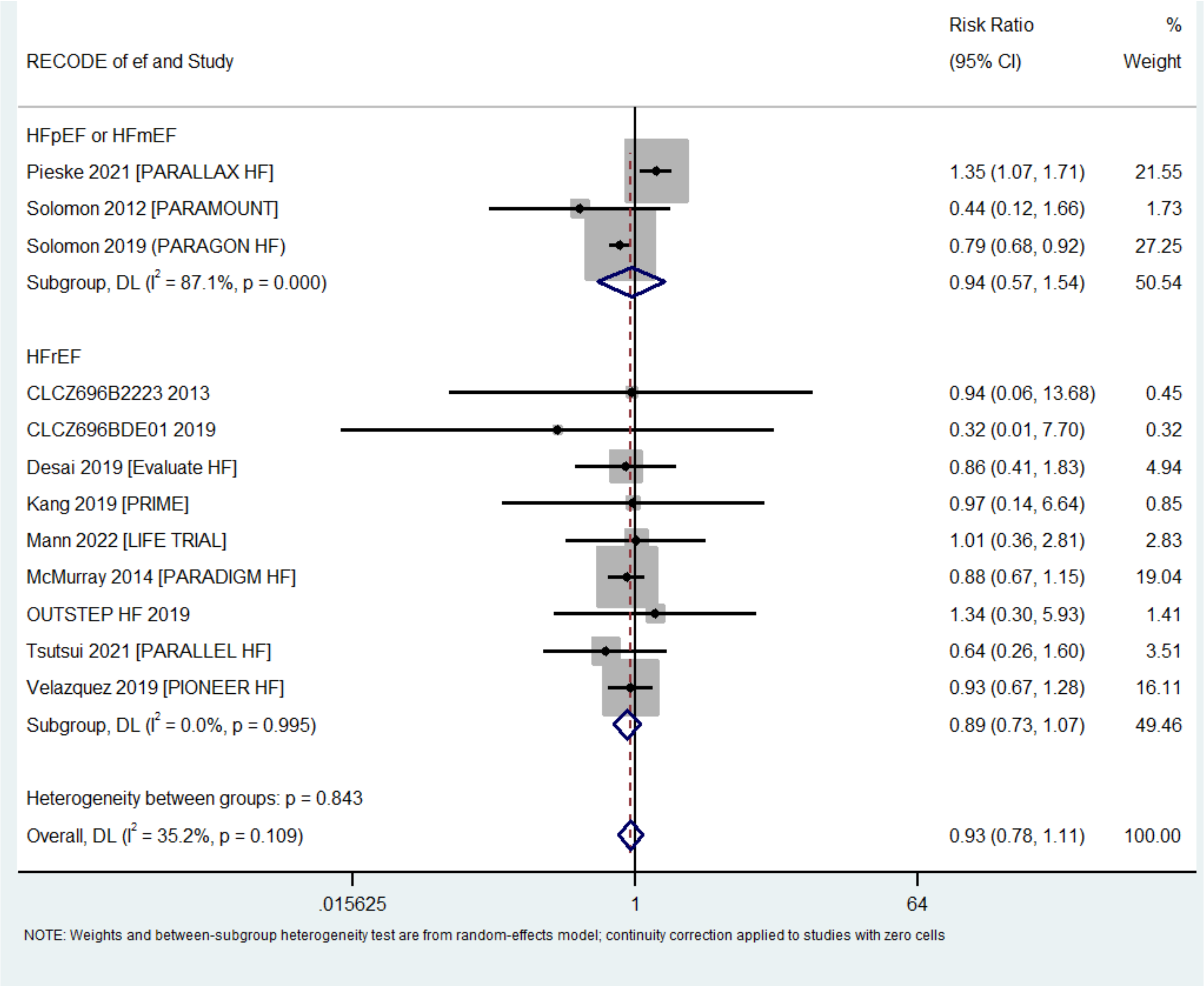

Supplementary Figure 19: Forest plot showing difference in worsening renal function between sacubitril-valsartan and control group based on type of comparison group

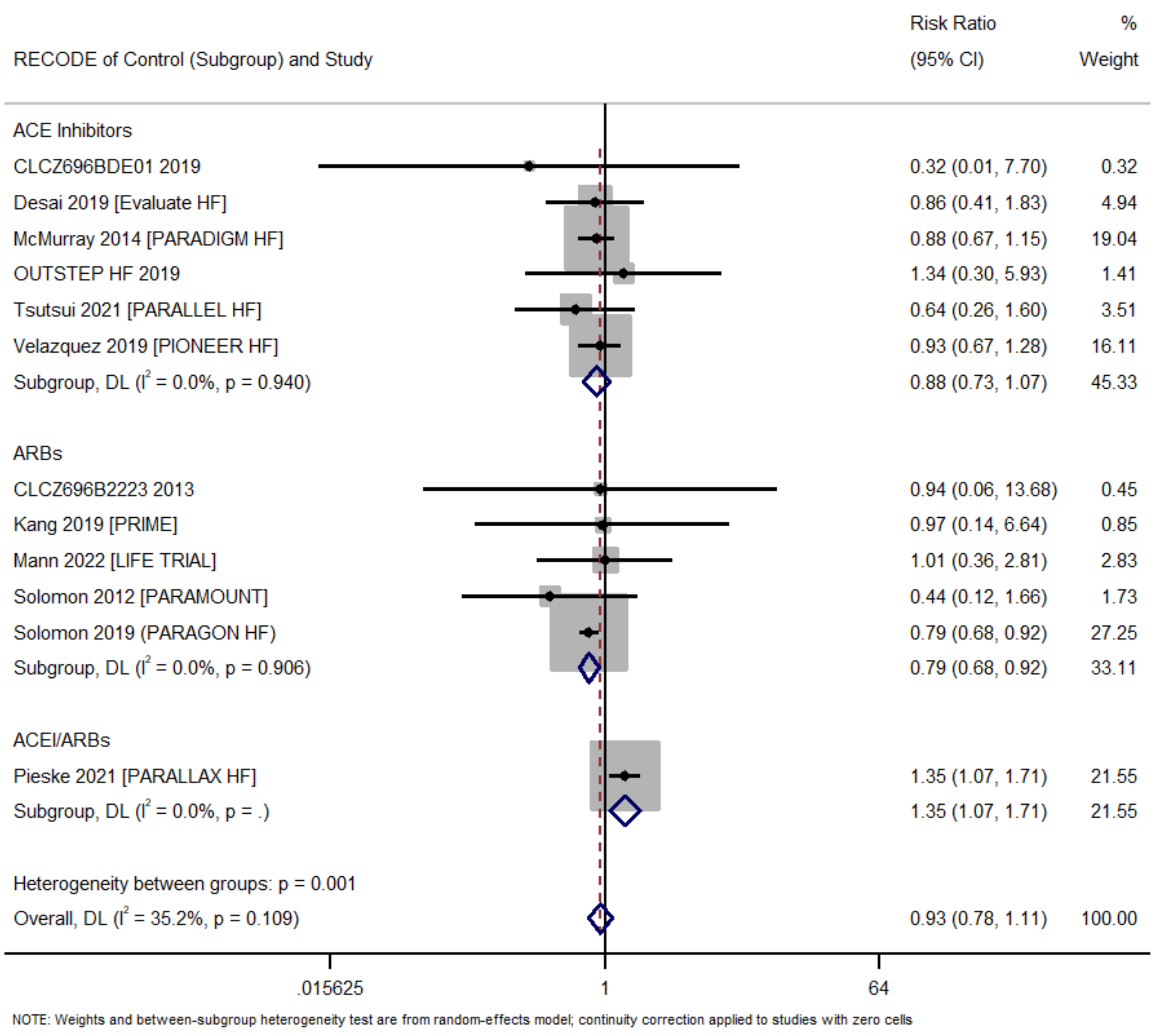

Supplementary Figure 20: Funnel plot for worsening renal function outcome

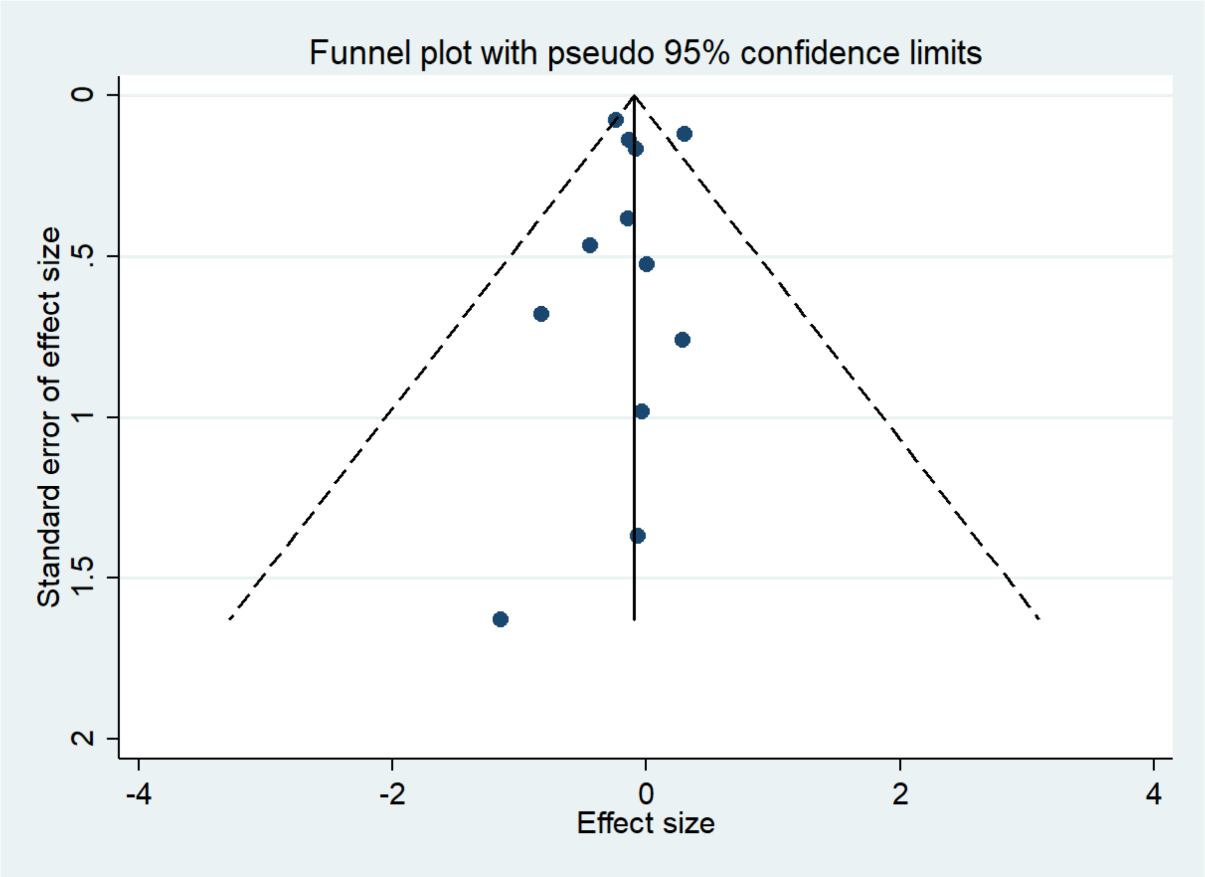

Supplementary Figure 21: Forest plot showing difference in hyperkalaemia between sacubitril-valsartan and control group based on status of ejection fraction

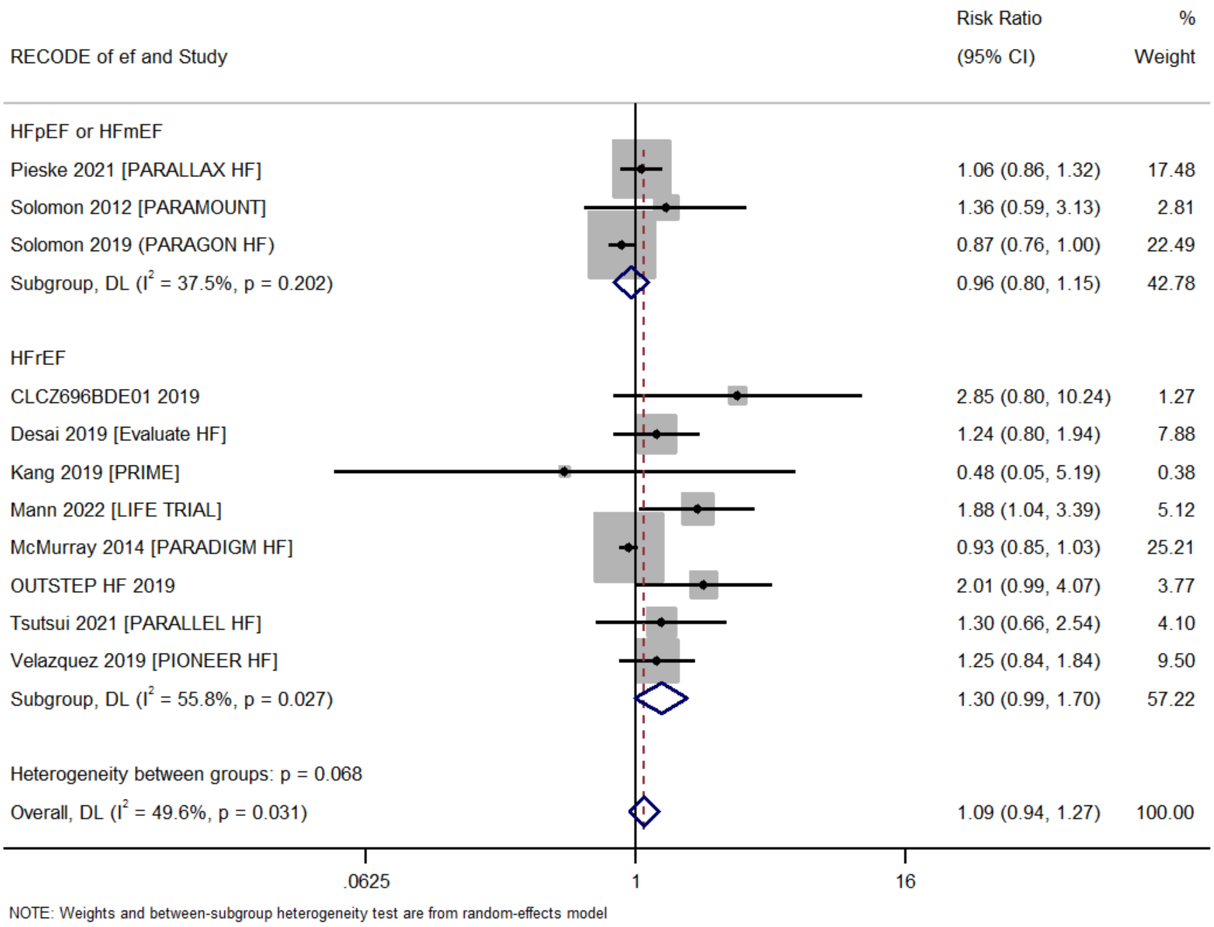

Supplementary Figure 22: Forest plot showing difference in hyperkalaemia between sacubitril-valsartan and control group based on type of comparison group

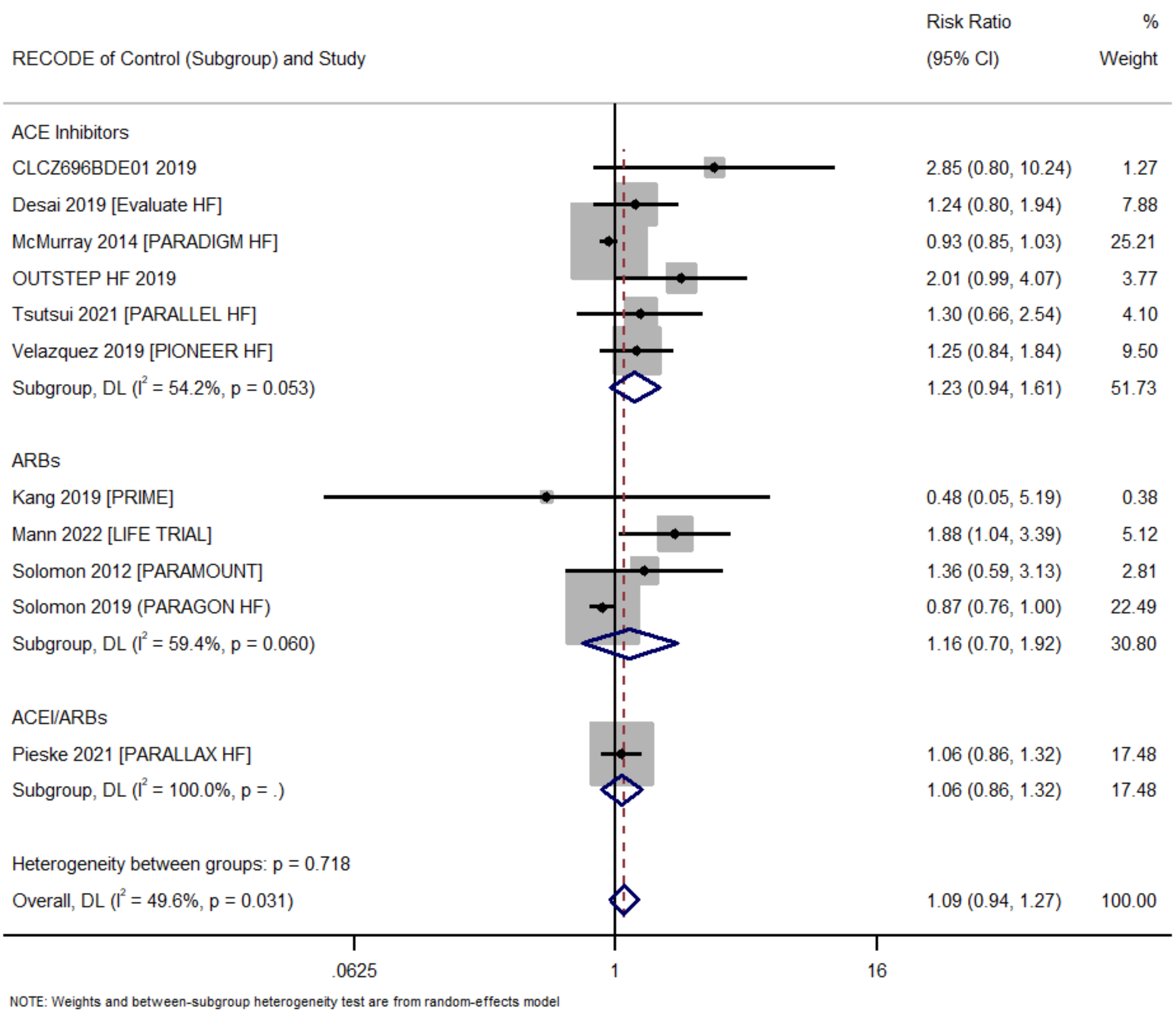

Supplementary Figure 23: Funnel plot for hyperkalaemia outcome

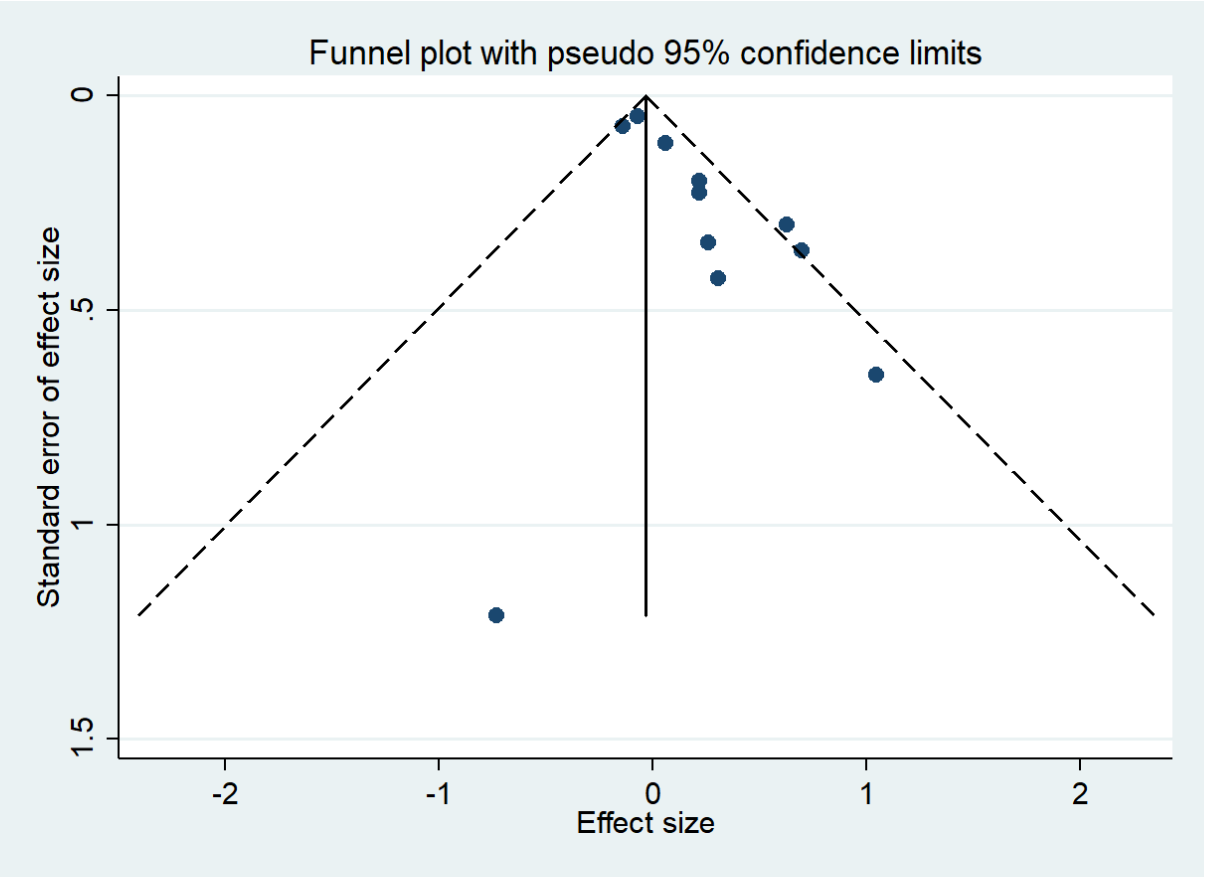

Supplement: Supplementary file 1 — Supplementary file1 (PDF 689 KB) [file 10741_2022_10273_MOESM1_ESM.pdf]
